# A Common East Asian aldehyde dehydrogenase 2*2 variant promotes ventricular arrhythmia with chronic light-to-moderate alcohol use in mice

**DOI:** 10.1038/s42003-023-04985-x

**Published:** 2023-06-06

**Authors:** An-Sheng Lee, Yen-Ling Sung, Szu-Hua Pan, Kuo-Tzu Sung, Cheng-Huang Su, Shiao-Li Ding, Ying-Jui Lu, Chin-Ling Hsieh, Yun-Fang Chen, Chuan-Chuan Liu, Wei-Yu Chen, Xuan-Ren Chen, Fa-Po Chung, Shih-Wei Wang, Che-Hong Chen, Daria Mochly-Rosen, Chung-Lieh Hung, Hung-I Yeh, Shien-Fong Lin

**Affiliations:** 1 Department of Medicine, MacKay Medical College, New Taipei, Taiwan; 2 Division of Cardiovascular Medicine, China Medical University Hospital, Taichung, Taiwan; 3 Institute of Biomedical Engineering, College of Electrical and Computer Engineering, National Yang Ming Chiao Tung University, Hsinchu, Taiwan; 4 Graduate Institute of Biomedical Optomechatronics, Taipei Medical University, Taipei, Taiwan; 5 Graduate Institute of Medical Genomics and Proteomics, College of Medicine, National Taiwan University, Taipei, Taiwan; 6 Genome and Systems Biology Degree Program, National Taiwan University and Academia Sinica, Taipei, Taiwan; 7 Doctoral Degree Program of Translational Medicine, National Taiwan University, Taipei, Taiwan; 8 Division of Cardiology, Departments of Internal Medicine, MacKay Memorial Hospital, Taipei, Taiwan; 9 Department of Medical Research, MacKay Memorial Hospital, New Taipei, Taiwan; 10 Department of Physiology Examination, MacKay Memorial Hospital, New Taipei, Taiwan; 11 Heart Rhythm Center and Division of Cardiology, Department of Medicine, Taipei Veterans General Hospital, Taipei, Taiwan; 12 Department of Medicine, National Yang Ming Chiao Tung University, School of Medicine, Taipei, Taiwan; 13 Institute of Biomedical Sciences, MacKay Medical College, New Taipei, Taiwan; 14 Department of Chemical and Systems Biology, Stanford University, School of Medicine, Stanford, CA USA

**Keywords:** Cardiology, Biomarkers

## Abstract

Chronic heavy alcohol use is associated with lethal arrhythmias. Whether common East Asian-specific aldehyde dehydrogenase deficiency (ALDH2*2) contributes to arrhythmogenesis caused by low level alcohol use remains unclear. Here we show 59 habitual alcohol users carrying *ALDH2* rs671 have longer QT interval (corrected) and higher ventricular tachyarrhythmia events compared with 137 *ALDH2* wild-type (Wt) habitual alcohol users and 57 alcohol non-users. Notably, we observe QT prolongation and a higher risk of premature ventricular contractions among human *ALDH2* variants showing habitual light-to-moderate alcohol consumption. We recapitulate a human electrophysiological QT prolongation phenotype using a mouse ALDH2*2 knock-in (KI) model treated with 4% ethanol, which shows markedly reduced total amount of connexin43 albeit increased lateralization accompanied by markedly downregulated sarcolemmal Nav1.5, Kv1.4 and Kv4.2 expressions compared to EtOH-treated Wt mice. Whole-cell patch-clamps reveal a more pronounced action potential prolongation in EtOH-treated ALDH2*2 KI mice. By programmed electrical stimulation, rotors are only provokable in EtOH-treated ALDH2*2 KI mice along with higher number and duration of ventricular arrhythmia episodes. The present research helps formulate safe alcohol drinking guideline for ALDH2 deficient population and develop novel protective agents for these subjects.

## Abstract (web summary)

A common variant in the aldehyde dehydrogenase, *ALDH2*, which is enriched in East Asian populations can promote the risk of ventricular arrhythmia in mice exposed to a low alcohol dose emulating light alcohol consumption in humans.

## Introduction

Despite the heavy burden imposed by cardiovascular diseases and under-recognized annual deaths among heavy alcohol drinkers and alcoholics^1^, the exact safe dose for alcohol consumption remains unclear and varies geographically^2,3^. The association between alcohol consumption and cardiovascular mortality appears to follow a J-curve^3^. A recent large-scale multi-ethnic epidemiological study showed that alcohol intake levels previously thought to be relatively harmless were associated with a higher risk of early death^4^. Prior experimental data and simulation models have linked alcohol-related arrhythmogenic effects to acute exposure to high doses of alcohol (blood alcohol concentration >20 mM) or chronic heavy drinking (≥6 drinks/day). Electrophysiological instability induced by exposure to alcohol, under the conditions stated above, is reportedly associated with dysregulated ion channel homeostasis, prolonged action potential, and re-entrant excitation, all of which result in increased vulnerability to ventricular arrhythmia (VA) and sudden cardiac death^5–7^. Currently, the exact toxic threshold of chronic alcohol intake and its mechanistic link to the development of VA, a serious yet uncommon life-threatening arrhythmia triggered by alcohol intake, remains a subject of controversy^8,9^.

Mitochondrial aldehyde dehydrogenase (ALDH2) plays a pivotal role in ethanol detoxification and cardioprotection against ischemia-reperfusion injury^10,11^. The *ALDH2* rs671 variant, or the ALDH2*2 allele, which encodes an inactive ALDH2 enzyme and causes the alcohol facial flushing reaction, is one of the most common and ethnicity-specific enzymes in humans, affecting ~540 million East Asians worldwide^12^. The ALDH2*2 variant has gained much attention recently due to its increased susceptibility to the toxic effects of alcohol^13,14^. A recent epidemiological analysis investigating dose-related cardiovascular complications caused by alcohol consumption indicated that the interpretation of these relationships may be biased due to a lack of sufficient information on genetic background^15^. We previously demonstrated that disturbed ventricular electrical conduction and re-entry were putative pro-arrhythmia mechanisms in animal high ethanol intake models^16^. We surmised that dysregulated electrophysiological features and re-entry mechanisms may cause VA with a lower alcohol dose, such as <2 standard drinks/day or moderate drinking, in humans showing ALDH2 deficiency. We conducted mechanistic experimental studies to explore the relationship between ALDH2 deficiency and VA under chronic low-to-moderate alcohol exposure using a mouse ALDH2*2 knock-in (KI) model. We also conducted a 5-year clinical longitudinal study to explore this relationship and the risk of arrhythmia among a cohort of 196 human subjects known for light-to-moderate alcohol consumption as well as the *ALDH2* rs671 genotype.

## Results

### Clinical episodes of VA and alcohol consumption from a 5-year follow-up study

#### Association of *ALDH2* genotype with alcohol consumption dose and baseline QTc

**Fig. 1:**
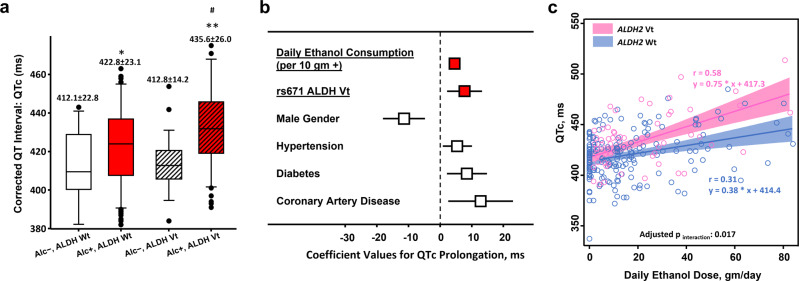
QT prolongation phenotype in *ALDH2* Vt alcohol users. *ALDH2* Vt alcohol users showed a significantly longer QT interval (corrected to RR interval as QTc) compared to alcohol non-users (Alc−) and *ALDH2* Wt alcohol users (430.1 vs 422.4 vs 415.6 ms in Alc−, Alc+ *ALDH2* Wt and Alc+ *ALDH2* Vt, respectively) (**a**). Box plot represents 25th and 75th percent quartiles, the line represents the median, and the whiskers are 10% (lower) and 90% (upper). * denotes *p* < 0.05 for Alc+ compared with Alc− within each genotype group (either *ALDH* Wt or *ALDH* Vt). Backward stepwise multi-variate regression models exploring the associations between clinical covariates and QTc interval in the current study (**b**). Higher daily alcohol intake amounts were associated with steeper QTc increments in subjects carrying *ALDH2* Vt (pink) versus *ALDH2* Wt (blue) (adjusted *p*_interaction_: 0.017) (**c**). Bars in **c** represent the standard deviation for QTc and the standard error for alcohol dose, respectively. The shade areas represent 95% confidence interval area. *p < 0.05, **p < 0.001 for Alc+ versus Alc− within each genotype category (either *ALDH* Wt or *ALDH* Vt); ^#^*p* < 0.05 for Alc+, *ALDH* Vt versus Alc+, *ALDH* Wt.

Among the 196 adults with habitual light-to-moderate alcohol consumption (median: 13.9 [7.6~29.3] g/day, 1.2 [0.6~2.4] standard drinks/day), 59 (30.1%) carried the *ALDH2* rs671 variant allele ([*ALDH2* Vt]; G/A or A/A), while 137 (69.9%) were *ALDH2* wild-type ([*ALDH2* Wt]; G/G, Table 1). Among the 57 alcohol non-users, 28 (49.1%) were *ALDH2* Vt and 29 (50.9%) were *ALDH2* Wt. These findings indicated that up to 70% of the study participants classified as habitual alcohol users were *ALDH2* Wt (*X*^2^ *p* = 0.008), whereas among the alcohol non-users, the distribution of wild-type and variant of *ALDH2* carriers was approximately equal (Table 1; Supplementary Fig. 1b), which approximated the same *ALDH2* genotype distribution (51% *ALDH2* Wt) found in the general, healthy Taiwanese population based on Taiwan Biobank data (https://taiwanview.twbiobank.org.tw). Overall, habitual alcohol users were more likely to be men regardless of *ALDH2* genotype and showed a higher prevalence of hypertension in *ALDH2* Wt group. Habitual alcohol users (Alc+) demonstrated more prolonged QRS duration and QTc compared to their respective alcohol non-users (Alc−) irrespective of *ALDH2* genotypes (both *p* < 0.05) (Table 1 and Fig. 1a), with habitual alcohol users (Alc+) carrying *ALDH2* Vt further showed significantly longer QTc compared to *ALDH2* Wt alcohol users (Alc+) (Fig. 1a). Backward multi-variate regression models showed that a higher dosage of alcohol consumption (adjusted coefficient: 4.52 [95% CI: 3.14–5.91], per 10 g+/day, *p* < 0.001), *ALDH2* Vt (adjusted coef: 7.67 [95% CI: 2.15–13.18], *p* = 0.003), female gender, history of hypertension, coronary artery disease (CAD), and diabetes were independently associated with prolonged QTc (Fig. 1b). Notably, *ALDH2* Vt individuals showed a higher, dose-dependent QTc prolongation slope (*r* = 0.58) compared with that of the *ALDH2* Wt participants (*r* = 0.31) at larger daily doses of alcohol intake (adjusted *p*_interaction_: 0.017) (Fig. 1c).

**Table 1:** Baseline characteristics and surface ECG information according to habitual alcohol consumption stratified by *ALDH2* genotypes.

	*ALDH2* Wt		*ALDH2* Vt (GA or AA genotype)	
	Alcohol non-users	Habitual alcohol users	Alcohol non-users	Habitual alcohol users
	Group 1 (*n* = 29)	Group 2 (*n* = 137)	Group 3 (*n* = 28)	Group 4 (*n* = 59)
Baseline demographics				
Age, years	50.4 ± 9.0	52.2 ± 10.4	53.2 ± 10.9	49.6 ± 9.0
Male gender	19 (65.5%)	116 (84.7%)	18 (64.3%)	50 (84.8%)
Body mass index, kg/m^2^	25.3 ± 2.9	25.9 ± 4.4	25.4 ± 4.0	25.9 ± 4.1
Hypertension, %	4 (13.8%)	72 (52.6%)	6 (21.4%)	18 (30.5%)
Diabetes, %	5 (17.2%)	30 (21.9%)	6 (21.4%)	20 (33.9%)
Hyperlipidemia treatment, %	0 (0%)	15 (11.0%)	1 (3.6%)	5 (8.5%)
CAD, %	0 (0%)	11 (8.0%)	0 (0%)	7 (11.9%)
eGFR, ml/min/1.73 m^2^	88.9 ± 16.2	92.6 ± 22.9	84.8 ± 20.3	94.8 ± 22.0
Alcohol intake amount				
Ethanol intake, gm/day^†^	0 [0 ~ 0]	14.2 [8.3 ~ 28.6]*	0 [0 ~ 0]	12.7 [6.3 ~ 32.8]*
Daily drinks, amount^†^	0 [0 ~ 0]	1.0 [0.6 ~ 2.0]*	0 [0 ~ 0]	0.9 [0.5 ~ 2.3]*
Electrocardiography (ECG)				
Heart rate, beats/min	66.3 ± 8.2	72.4 ± 16.2	68.1 ± 12.5	72.5 ± 12.9
QRS duration, ms	86.0 ± 9.2	92.8 ± 9.7*	86.9 ± 10.0	96.3 ± 10.5*

*CAD* coronary artery disease. ^†^median [IQR: 25th~75th].

*p < 0.05 for habitual alcohol users versus alcohol non-users within each genotype category (*ALDH2* Wt or Vt).

#### Association of ALDH2*2 variant with VAs development

Serial follow-up ECGs and a symptom-driven Holter study were used to grade the clinical frequency and severity of VAs in our study participants. Seventeen study participants with VAs (~7%) were identified during a 5-year follow-up period. None were found in non-alcohol users among the participants, whereas 6 (4.4%) were detected in *ALDH2* Wt habitual alcohol users, with habitual alcohol users carrying *ALDH2* Vt showing a nearly 4-fold higher risk for developing significant VAs (*n* = 11, 18.6%) (*X*^2^ *p* < 0.001). Multi-variate logistic regression models used to examine the predictors of VAs are displayed (Table 2). Apart from a known history of CAD, individuals carrying *ALDH2* Vt had a four times higher risk (adjusted odds ratio: 4.46, 95% CI: 1.50–13.23, *ALDH2* Wt as reference) for developing VA episodes. A higher dosage of alcohol consumption was independently associated with a higher risk of significant VAs (adjusted odds ratio: 1.41, 95% CI: 1.13–1.74, per 10 g/day increment; Table 2), the risk being more pronounced in *ALDH2* Vt carriers (adjusted *p*_interaction_: 0.038); (Table 2). The presence of *ALDH2* Vt (rs671) did not affect CAD history in terms of VA development (adjusted *p*_interaction_: 0.30). When Youden’s index was used for optimal cut-point estimation, the daily ethanol intake threshold for significant VAs in study participants of *ALDH2* Vt and *ALDH2* Wt was 9.6 g/day (0.7 drinks/day) and 14.1 g/day (1.0 drink/day), respectively.

**Table 2:** Predictors of VAs in stepwise multivariate logistic regression models.

Variables	Odds ratio	[95% confidence interval]	*p* value	*p*_interaction_ x rs671^†^
rs671 ALDH2*2 Vt (GA or AA genotype)	4.46	1.50−13.23	0.01	—
Alcohol Consumption (per 10 g+/day)	1.41	1.13−1.74	0.002	0.038
CAD	4.98	1.29−19.29	0.02	0.30

^†^Model adjusted for age, sex, BMI, and clinical comorbidities including hypertension, diabetes, CAD, hyperlipidemia treatment, and renal function in terms of eGFR.

#### Alcohol-induced VA in an ALDH2*2 KI mouse model

##### Electrocardiogram (ECG) recordings

**Fig. 2:**
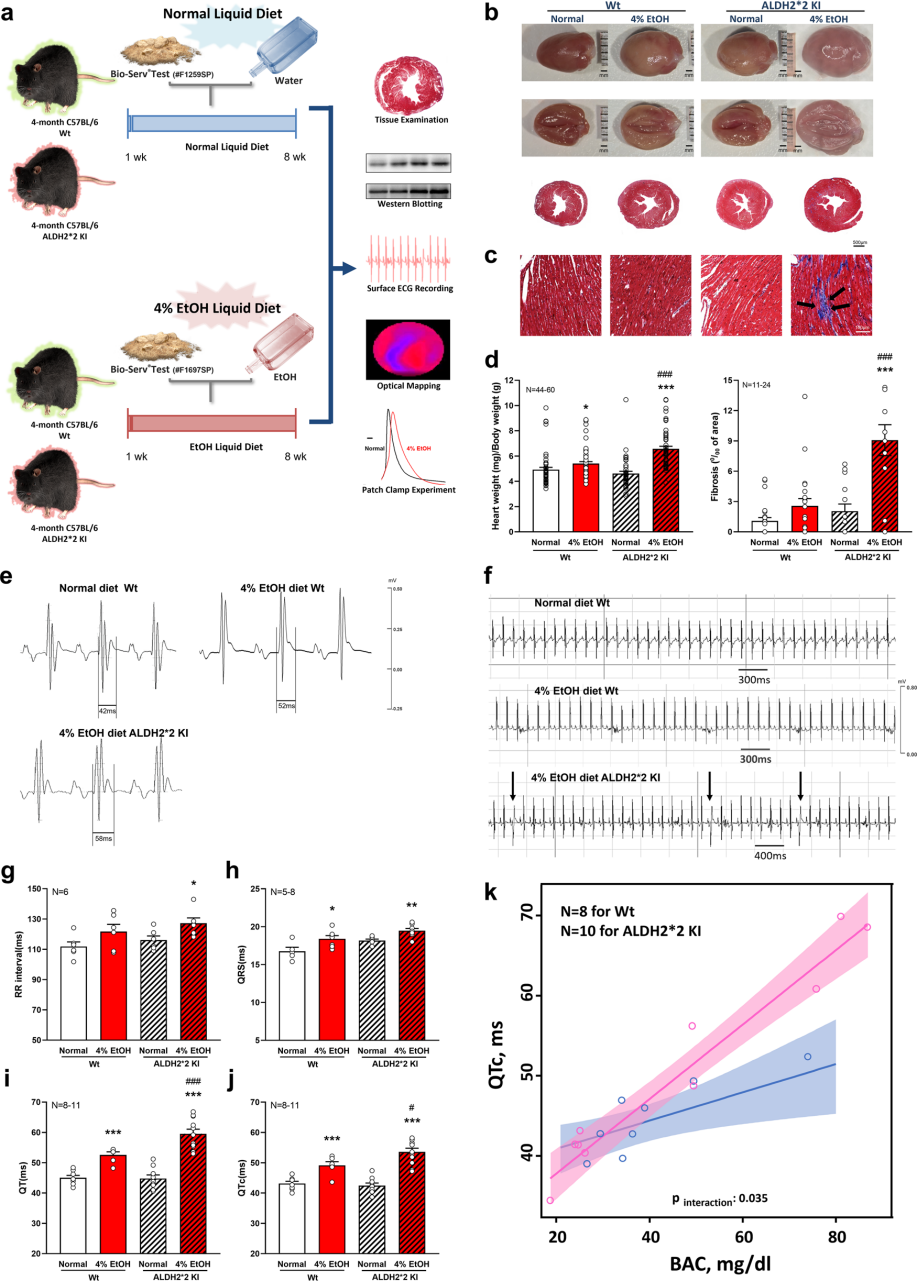
Schematic of the establishment and generation of the EtOH-treated ALDH2 deficiency (ALDH2*2 KI) model. Four-month-old homozygous ALDH2*2 KI mice (C57BL/6, male) after 7 weeks of 4% Alc diet treatment (**a**). Pathology-related findings in representative gross morphology and cross-sections of mice hearts from different groups showing preserved yet slightly higher myocardial mass and LV wall thickness in EtOH-treated ALDH2*2 KI mice (**b**). Histological analysis (H&E) with Masson’s trichrome (MT) staining of heart sections (**c**). Heart weight indexed to body weight of mice revealed a slight but significant increase in 4% EtOH-treated mice groups (left), particularly in ALDH2*2 KI mice, compared to those of their respective normal diet control groups (**d**). Markedly extensive fibrosis (black arrows) within the myocardium (light blue staining) was also observed in EtOH-treated ALDH2*2 KI mice (right) (**d**). Illustrations of body surface electrocardiogram (ECG) in the mouse model. 4% EtOH-treated ALDH2*2 KI mice showed electrocardiographic phenotypes of more prolonged QRS duration, QT interval (**e**), and spontaneous onset of frequent VPCs (black arrow) (**f**) compared to other groups during 5 min of continuous ECG recording. On average, QRS and QTc showed 16% and 23.6% increases compared to normal diet Wt mice in EtOH-treated ALDH2*2 KI mice (both *p* < 0.05) by surface ECG (**g**–**j**). Higher QTc prolongation slope in 4% EtOH-treated ALDH2*2 KI mice (pink) compared to that of the 4% EtOH-treated Wt mice (blue) at higher BAC levels (*p*_interaction_: 0.035) was observed (Fig. 2k). The shade areas represent 95% confidence interval area. Error bars represent standard error. *p < 0.05, **p < 0.01, ***p < 0.001 for any 4% EtOH vs normal diet control within each genotype group (ALDH2*2 KI or Wt); ^#^*p* < 0.05, ^##^*p* < 0.01, ^###^*p* < 0.001 for 4% EtOH ALDH2*2 KI vs 4% EtOH Wt.

The experimental protocol is shown (Fig. 2a). The blood alcohol concentration (BAC) in wild-type (Wt) and ALDH2*2 KI mice fed normal and 4% EtOH liquid diets for 7 weeks was determined (Supplementary Fig. 2). Both Wt and ALDH2*2 KI mice treated with 4% alcohol diet for 7 weeks demonstrated a slight increase in the gross morphology of heart wall thickness and heart size, compared to the groups without alcohol diet (Fig. 2b). More extensive myocardial interstitial fibrosis was observed in the 4% EtOH-treated ALDH2*2 KI mice compared to that of 4% EtOH-treated Wt mice and other groups (Fig. 2c, d). ALDH2*2 KI mice fed 4% EtOH also showed a significantly increased heart weight/body weight (HW/BW) ratio compared to that of 4% EtOH-treated Wt mice and other groups (Fig. 2d). The BACs were 0.011 ± 0.001 vol% (2.120 ± 0.281 mM), 0.057 ± 0.007 vol% (9.443 ± 1.102 mM), 0.013 ± 0.002 vol% (2.098 ± .0.270 mM) and 0.068 ± 0.007 vol% (11.58 ± 1.171 mM) for Wt/normal diet, Wt/4% EtOH diet, ALDH2*2 KI/normal diet, and ALDH2*2 KI/4% EtOH diet mice groups, respectively (Supplementary Fig. 2). Notably, the electrocardiographic phenotype of 4% EtOH-treated ALDH2*2 KI mice replicated the human phenotype showing marked prolongation of QT intervals (QT and QTc) and spontaneous onset of VAs as compared to the 4% EtOH-treated Wt mice and other groups (2 out of 7 mice vs. none in other mice groups, Fig. 2e–g, j). Similar trend on higher QTc prolongation slope in 4% EtOH-treated ALDH2*2 KI mice compared to that of the 4% EtOH-treated Wt mice at higher BAC levels (*p*_interaction_: 0.035) (Fig. 2k) was observed.

##### Perturbed connexin43 (Cx43) and ion channel protein expression in ALDH2*2 KI mice treated with light-to-moderate alcohol

**Fig. 3:**
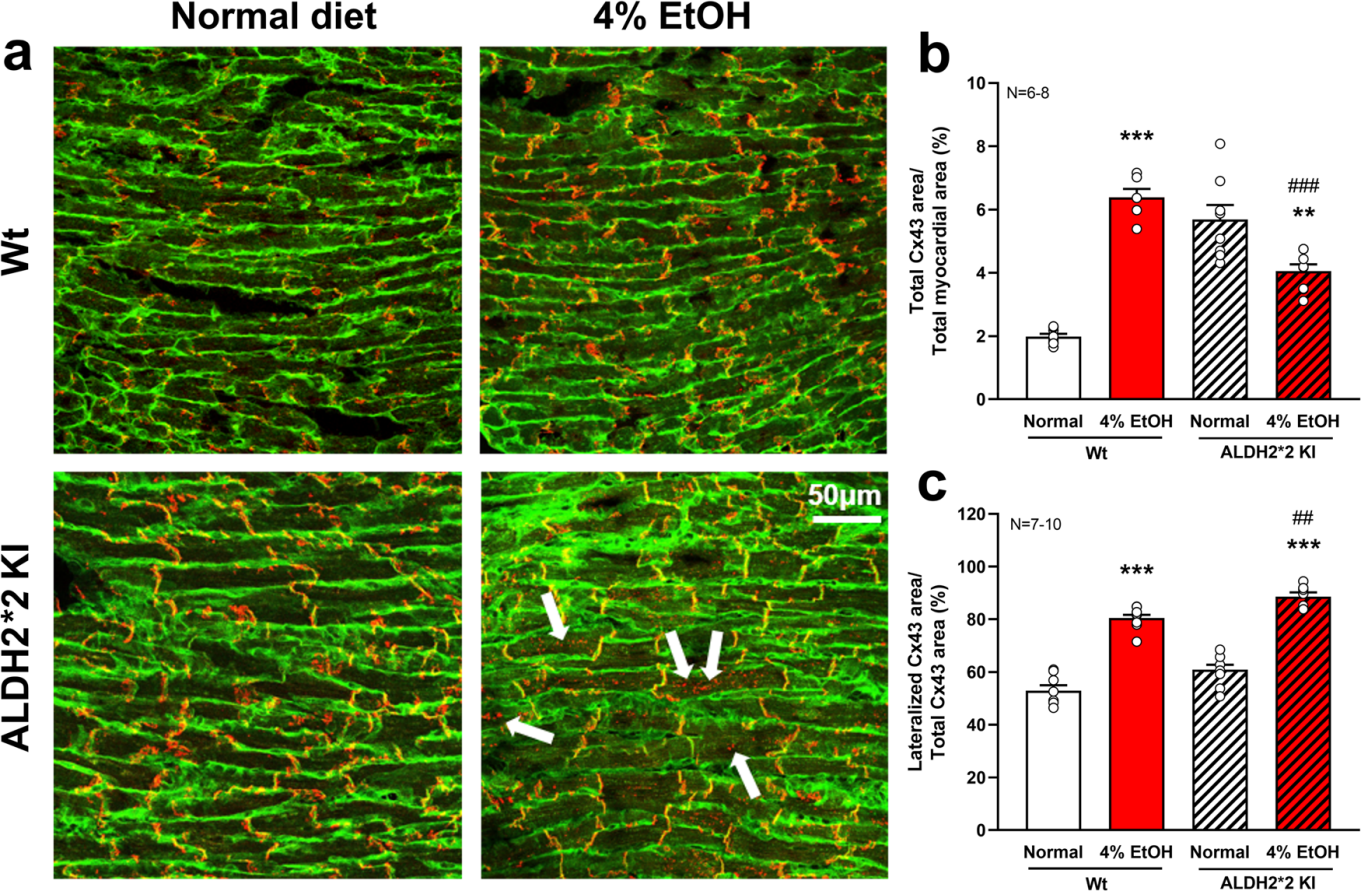
Expression of Cx43 in cardiomyocytes by immuno-confocal microscopy. Immuno-confocal microscopy for myocardial double staining for WGA and Cx43 showed a significantly reduced total Cx43 expression area (with and without total myocardial area correction) in EtOH-treated ALDH2*2 KI mice compared to that of the EtOH-treated Wt and normal diet ALDH2*2 KI mice groups, with a featured increase in cytoplasmic Cx43 staining (white arrows) (**a**, **b**). A markedly increased ratio of Cx43 redistribution on lateral cardiomyocyte borders was observed in both EtOH-treated mice groups when compared to that of the other two normal diet control groups (**a**, **c**), with EtOH-treated ALDH2*2 KI mice exhibiting more pronounced increase of Cx43 lateralization than EtOH-treated Wt mice. For each group, 13–15 images were analyzed (3 mice in each group) (×40, 800 pixels). Error bars represent standard error. *p < 0.05, **p < 0.01, ***p < 0.001 for any 4% EtOH vs normal diet control within each genotype group (ALDH2*2 KI or Wt); ^#^*p* < 0.05, ^##^*p* < 0.01, ^###^*p* < 0.001 for 4% EtOH ALDH2*2 KI vs 4% EtOH Wt.

Next, we examined the gap junction protein, Cx43, and several key ion channels/transporters involved in ventricular depolarization/repolarization. Double staining of myocardial tissues with Cx43 antibody revealed that Cx43 was upregulated in 4% EtOH-treated Wt mice as compared to the normal diet Wt mice (Fig. 3a, b). By contrast, Cx43 was significantly downregulated in EtOH-treated ALDH2*2 KI mice as compared to that of the EtOH-treated Wt mice (36.6% reduction, *p* = 0.006. Figure 3b). Cx43 distribution in the cardiomyocytes of both EtOH-treated mice was altered. Although Cx43 was predominantly confined to the intercalated discs in normal diet Wt mice, a higher ratio of Cx43 labels was observed at the lateral edges of the cardiomyocytes in both EtOH-treated mice groups. These findings indicated enhanced Cx43 lateralization in both EtOH-treated Wt and ALDH2*2 KI groups compared to that of their respective normal diet control groups, with EtOH-treated ALDH2*2 KI mice showing more pronounced Cx43 lateralization compared to that of the EtOH-treated Wt mice (52.8% and 67.2% increase in EtOH-treated Wt and ALDH2*2 KI mice groups versus normal diet Wt mice, respectively, both *p* < 0.001) (Fig. 3a, c).

The morphological analysis of various ion channel protein expressions among different mouse groups via immunofluorescence microscopy are illustrated in Supplementary Fig. 3. Ventricular cardiomyocytes were stained for Nav1.5 (green), Cav1.2 (green), Cav1.3 (green), Kv1.4 (green), Kv4.2 (green), Kv4.3 (green), and WGA (red), along with DAPI staining (blue). In brief, the immunofluorescence microscopy indicated that compared with normal diet Wt mice, the cardiomyocytes of both EtOH-treated mice showed morphological alterations and ion channels remodeling as well as abnormal architectural cardiomyocyte striation patterns, which were more prominent in ALDH2*2 KI mice. These may include disorganized staining of Nav1.5 (white arrowhead), attenuated staining and loss of striation of Kv1.4 (white arrowhead and asterisk labeled), irregular aggregations and attenuated staining of Kv4.2 (white arrowhead and asterisk labeled), accentuated staining of Kv4.3 (white arrowhead) and both accentuated Cav1.2 and Cav1.3 though altered aggregation in Cav1.2 (white arrowhead).

**Fig. 4:**
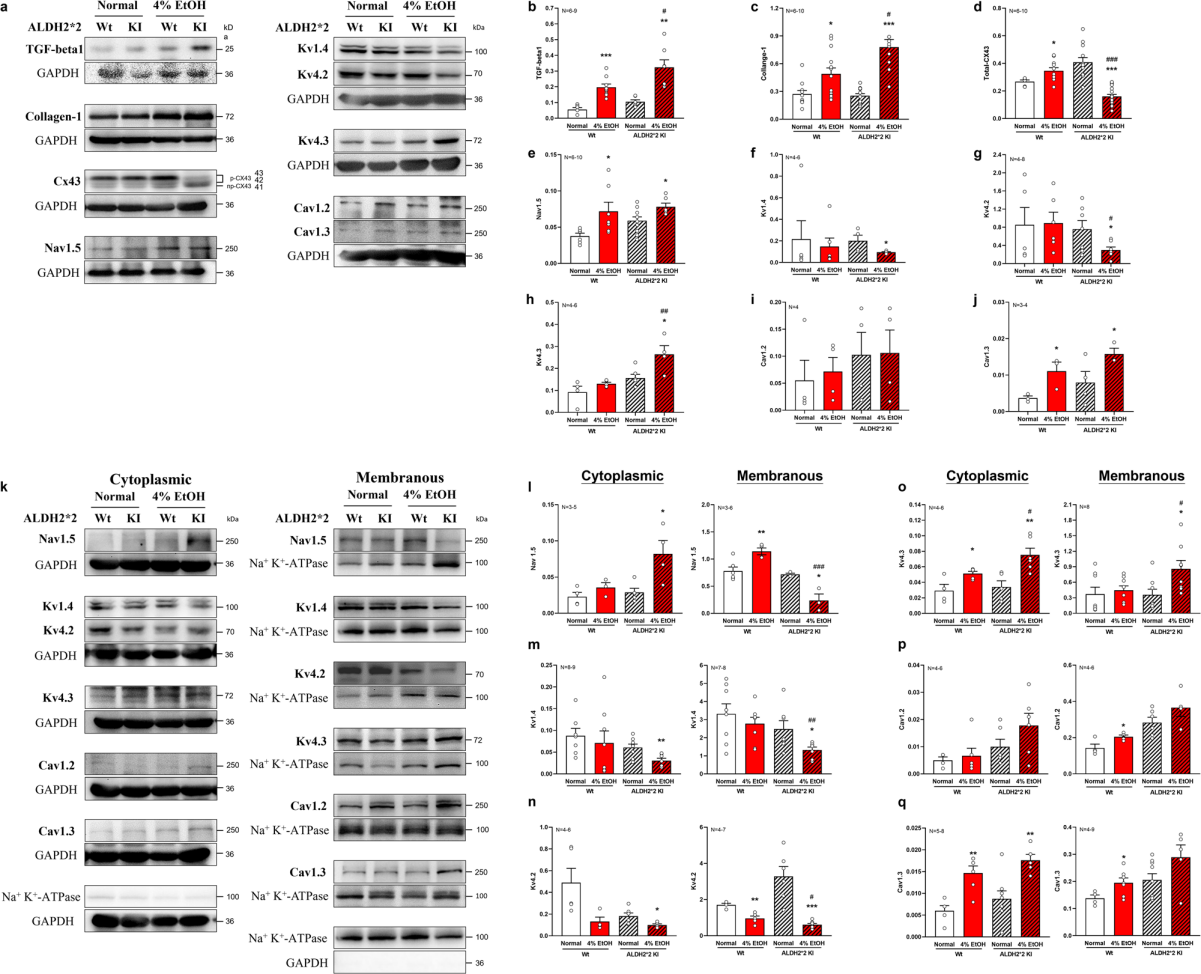
Western blotting analysis of heart tissue. Densitometric analysis of heart tissue TGF‐β1/collagen-1, Cx43 (total form), and a variety of ion channel proteins (including Nav1.5, Kv1.4, Kv4.2, Kv4.3, Cav1.2, and Cav1.3) of four groups of mice (**a**–**q**) via western blotting. Compared to normal diet Wt mice, EtOH-treated ALDH2*2 KI mice showed a 40.4% reduction in total Cx43, a 74.3% reduction in Kv1.4 and a 67.2% reduction in Kv4.2 (**d**, **f**, **g**), respectively. Cytoplasmic and membranous fractions of ion channel expression levels (**k**–**q**) were separately examined to determine the subcellular distribution of their alterations in distinctly ALDH2 functioning mice fed normal or 4% EtOH diet. Error bars represent standard error. *p < 0.05, **p < 0.01, ***p < 0.001 for any 4% EtOH vs normal diet control within each genotype group (ALDH2*2 KI or Wt); ^#^*p* < 0.05, ^##^*p* < 0.01, ^###^*p* < 0.001 for 4% EtOH ALDH2*2 KI vs 4% EtOH Wt.

We performed quantitative western blotting experiments to quantitate protein expression (Fig. 4a–q). Western blotting of whole-tissue preparations showed upregulated TGF‐β1 and collagen-1 in the myocardium of both EtOH-treated Wt and ALDH2*2 KI mice. Again, TGF‐β1 and collagen-1 were more prominently expressed in EtOH-treated ALDH2*2 KI mouse hearts (Fig. 4b, c). Cx43 total protein expression was significantly downregulated in EtOH-treated ALDH2*2 KI compared to the EtOH-treated Wt mice and other groups (all *p* < 0.05) (Fig. 4d). By contrast, Cx43 total protein expression in EtOH-treated Wt mice was upregulated compared to that in normal diet-treated Wt mice. Ion channel expression levels involved in ventricular depolarization and repolarization were reflected by the total expression of Nav1.5 expression which was slightly upregulated in both EtOH-treated Wt and ALDH2*2 KI mice as compared to that of their respective normal diet control groups (*p* = 0.034 and 0.028, respectively) (Fig. 4e). Both Kv1.4 and Kv4.2 were significantly downregulated in EtOH-treated ALDH2*2 KI mice compared to those of normal diet ALDH2*2 KI control mice groups (*p* = 0.029 and 0.039, respectively), with significantly lower Kv4.2 expression observed in EtOH-treated ALDH2*2 KI mice compared to that of the EtOH-treated Wt mice. These differences were not significant in EtOH-treated Wt mice versus those normal diet Wt mice (*p* = 0.71 and 0.93) (Fig. 4f, g). Kv4.3 was substantially upregulated in EtOH-treated ALDH2*2 KI mice compared to that of the EtOH-treated Wt mice and other groups (Fig. 4h). L-type Cav1.3 was significantly upregulated in EtOH-treated Wt mice compared to the normal diet Wt mice group (*p* = 0.044) though no significant differences for Cav1.2 were found between groups (Fig. 4i, j). Both sodium-calcium exchanger (NCX) and CaMKII (total expression and their phosphorylated/oxidized forms) were upregulated in EtOH-treated compared to those of their respective normal diet control groups irrespective of genotypes, with CaMKII being the most prominently upregulated protein in EtOH-treated ALDH2*2 KI mice (Supplementary Fig. 4).

As the membrane fraction of ion channels remain the fundamental functioning component of electrical impulse and action potential generation, we further examined the myocardial protein expression levels of the membranous fraction, separately from those of the cytoplasmic fraction (Fig. 4k–q). We observed distinctively regulated Nav1.5 expressions in EtOH-treated mice. Sarcolemmal Nav1.5 was markedly downregulated in EtOH-treated ALDH2*2 KI mice compared to that of EtOH-treated Wt mice (*p* < 0.001); by contrast, EtOH treatment led to upregulated sarcolemmal Nav1.5 in Wt mice compared to the normal diet Wt mice (*p* = 0.008) (Fig. 4l). Both sarcolemmal Kv1.4 and Kv4.2 showed marked downregulation in EtOH-treated ALDH2*2 KI mice when compared to those of their respective EtOH-treated Wt mice groups (both *p* < 0.05, respectively) (Fig. 4m, n). Conversely, membranous Kv4.3 in EtOH-treated ALDH2*2 KI mice was significantly upregulated compared to that of the EtOH-treated Wt mice and other groups (Fig. 4o). L-type calcium channels of Cav1.2 and Cav1.3 in the membranous fraction were significantly upregulated in EtOH-treated Wt mice compared to their normal diet Wt control mice, though these differences were not observed in ALDH2*2 KI mice groups. Finally, significant upregulations of cytoplasmic Cav1.3 but not Cav1.2 were observed in both EtOH-treated mice groups compared to that of their respective normal diet controls irrespective of genotypes (Fig. 4p, q).

##### Optical mapping and simulation of VA

**Fig. 5:**
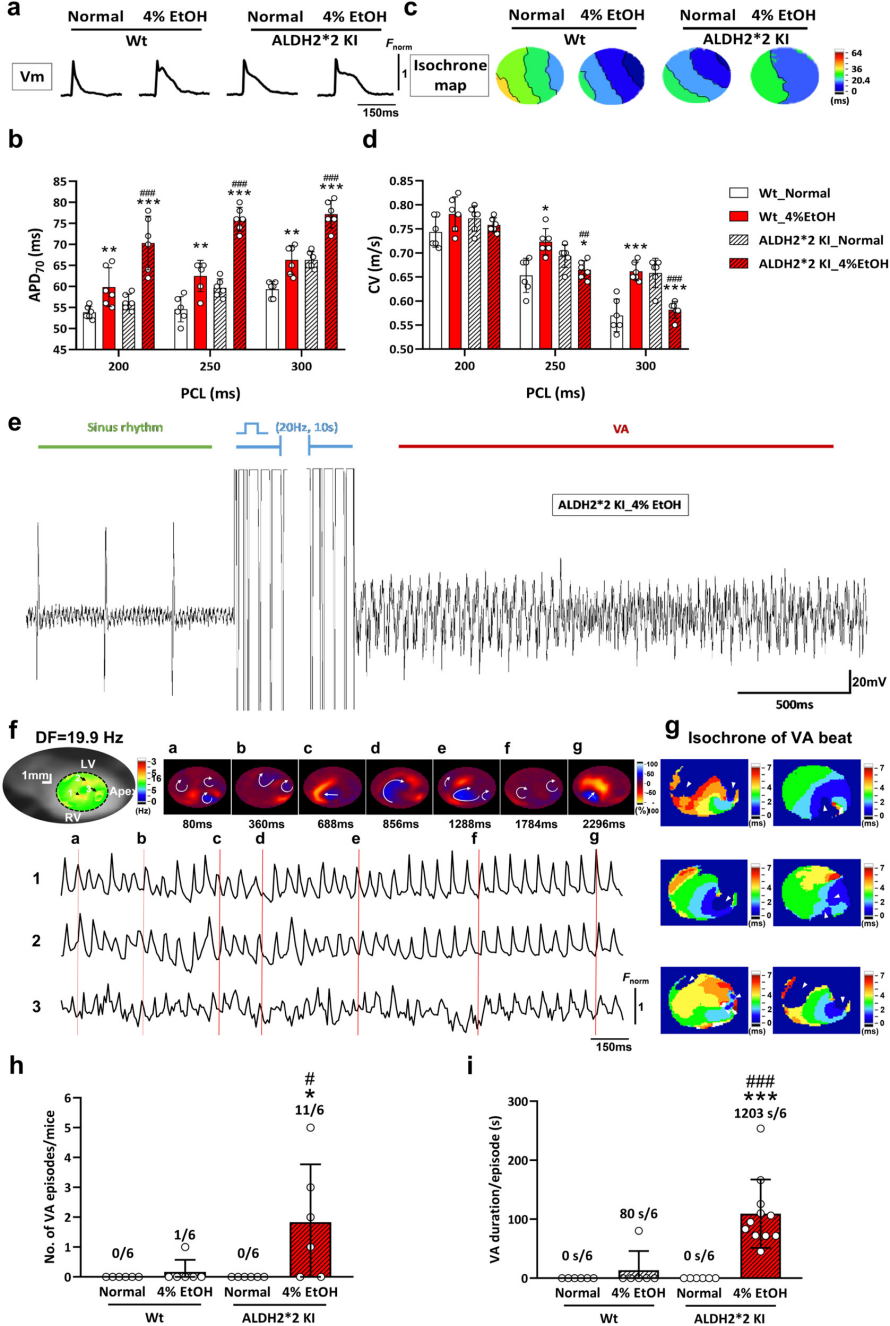
Findings on programmed electrical stimulation induction at different pacing cycle lengths. **a** Optical action potential at 300 ms PCL. **b** APD at 70% repolarization. **c** An example of electrical propagation as isochrone maps in the ventricle at 300 ms PCL. **d** CV at different PCLs; *n* = 6 in each group; error bars are SD; *p* values were calculated using GraphPad Prism 8.0 with multiple *t* tests. *APD* action potential duration, *CV* conduction velocity, *PCL* pacing cycle length. **e** Simultaneous ECG recordings and ventricular optical mapping were performed during VA induction from an example of malignant VA in EtOH-treated ALDH2*2 KI mice. EtOH-treated Wt mice showed only one episode of VA with a slight wave break and failed to exhibit rotations during VA (Supplementary Fig. 2, Supplementary Movie 2). Wt and ALDH2*2 KI mice fed normal diet revealed smooth electrical propagation following PES induction (Supplementary Movies 3 and 4). **f** Dominant-frequency (DF) points and snapshots of the spiral wave corresponding to optical signal in time domain at a given point during VA with **g** multiple VA beats and initiation sites shown (white arrows). **h**, **i** Quantification of induced VA episodes and duration. (*n* = 6 in each group). Error bars represent standard deviation; *p* values were calculated via unpaired Student’s *t* tests using GraphPad Prism 8.0. *VA* ventricular arrhythmia, *PES* programmed electrical stimulation, *DF* dominant frequency.

Dynamic changes in action potential duration (APD) and conduction velocity (CV) were analyzed to evaluate the effects of alcohol on cardiac electrical propagation patterns of both WT and ALDH2*2 KI mice. The membrane potential in the left ventricle epicardial surface assessed via an optical recording at a pacing cycle length (PCL) of 300 ms is shown (Fig. 5a). Mean APD_70_ was consistently longer in EtOH-treated mice compared to those of their respective normal diet control groups regardless of genotypes, with overall significantly higher APD_70_ observed in EtOH-treated ALDH2*2 KI mice compared to that of the EtOH-treated Wt mice (Fig. 5b). There were no significant differences between the CVs of the four different groups at a PCL of 200 ms. Despite significantly increased CVs in EtOH-treated mice compared to that in normal diet Wt mice, we observed that CVs were substantially decreased in EtOH-treated mice compared to that of the normal diet ALDH2*2 KI mice at PCLs of 250 and 300 ms. EtOH-treated ALDH2*2 KI mice further showed significantly lower CVs compared to those of the EtOH-treated Wt mice at PCLs of 250 and 300 ms (Fig. 5c, d). These results indicated that EtOH-treated ALDH2*2 KI mice exhibited significantly prolonged APD and lower CVs at higher PCLs.

Susceptibility of EtOH-treated ALDH2*2 KI mice to arrhythmia was further tested via programed stimulation. During VA induction, high vulnerability of malignant VAs along with inducible high-frequency re-entrant activity was observed in EtOH-treated ALDH2*2 KI mice after PES (11 episodes/6 mice in EtOH-treated ALDH2*2 KI vs 0–1 episode/6 mice in other groups, all *p* < 0.05) (Fig. 5e–i; Supplementary Figs. 5, 6). Using a dominant frequency map, high-frequency areas of VA rotations and inducible meandering rotors with multiple disorganized wavelets were observed in 4 out of 6 EtOH-treated ALDH2*2 KI mice during VA beats, but not in other groups (Fig. 5f, g; Supplementary Figs. 5, 6; Supplementary Movie 1). In comparison, only one EtOH-treated Wt mouse showed abnormal activation wave fronts with inducible VAs (Supplementary Fig. 6b; Supplementary Movie 2), and none of the mice in either the normal diet Wt or the normal diet ALDH2*2 KI group showed inducible VA (Supplementary Figs. 6a, c; Supplementary Movies 3, 4). EtOH-treated ALDH2*2 KI mice had significantly higher number and duration of VA episodes compared to those of the EtOH-treated Wt mice (Fig. 5h, i). These results demonstrated that increased VA episodes as well as VA durations recapitulated the main pro-arrhythmogenic features in EtOH-treated ALDH2*2 KI mice.

##### Patch clamp measurement of APD in single cardiomyocytes

**Fig. 6:**
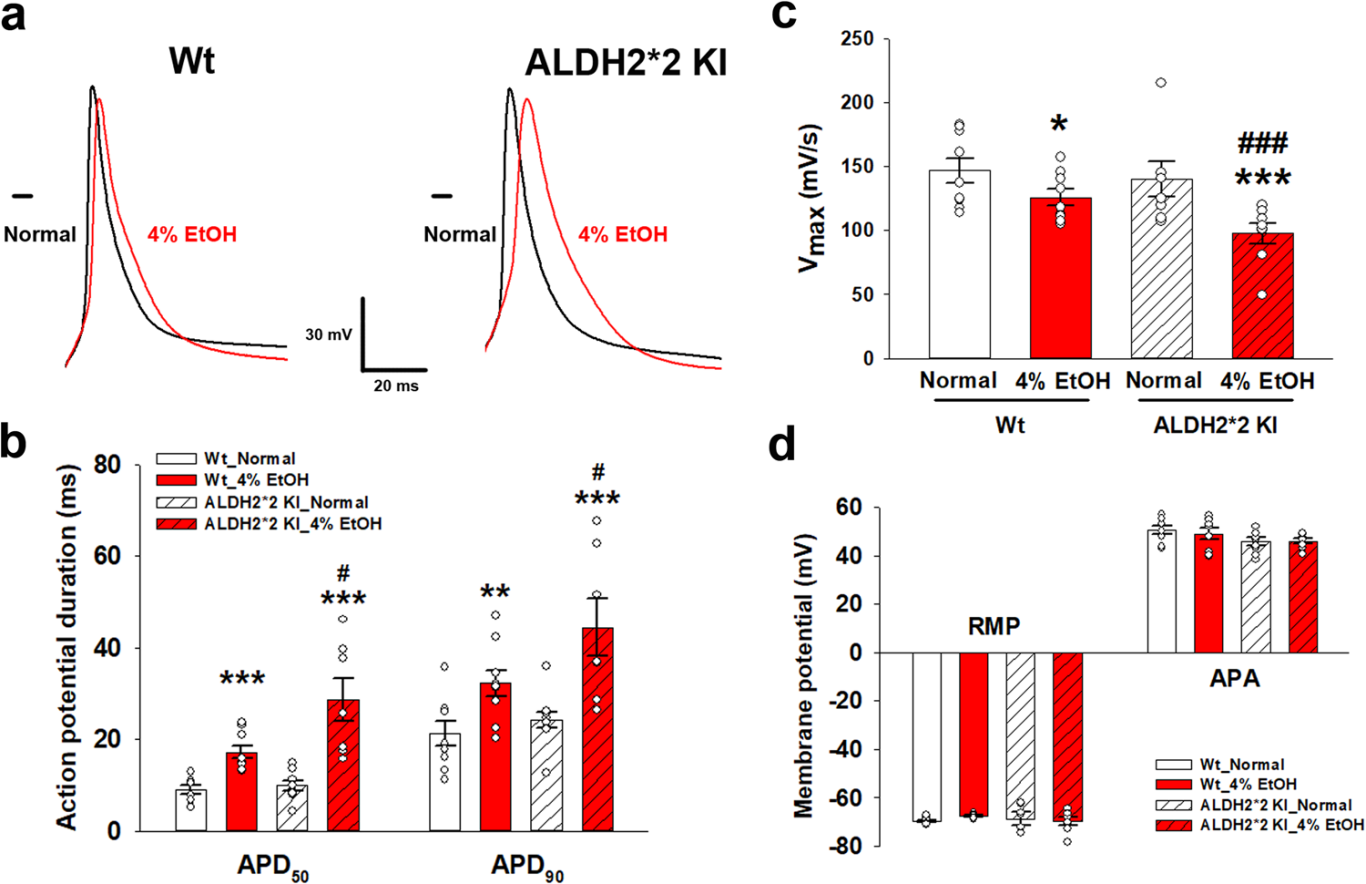
Determination of action potential duration (APD) and maximal upstroke velocity of action potential (*V*_max_) in the cardiomyocytes. Comparisons of superimposed recordings of APD at 50% and 90% repolarization (**a**, **b**), *V*_max_ (**c**), resting membrane potential (RMP), and action potential amplitude (APA) **d** among different mouse groups. *V*_max_ was 97.6 ± 8.0 vs. 140.4 ± 13.9 mV/s for EtOH-treated and normal diet ALDH2*2 KI mice, respectively; and 125.9 ± 6.3 vs. 146.9 ± 9.6 9 mV/s for EtOH-treated and normal diet Wt mice, respectively. *p < 0.05, **p < 0.01, ***p < 0.001 for any 4% EtOH vs normal diet control within each genotype group (ALDH2*2 KI or Wt); ^#^*p* < 0.05, ^##^*p* < 0.01, ^###^*p* < 0.001 for 4% EtOH ALDH2*2 KI vs 4% EtOH Wt. *n* = 8 independent cells in each group. Error bars represent standard error.

To determine whether APD of ALDH2*2 KI mice treated with EtOH is altered, isolated cardiomyocytes were monitored using the patch clamp technique. Cardiomyocytes were paced with 5 ms suprathreshold depolarizing stimuli in the current-clamp mode. APD (at 50% and 90% repolarization) was prolonged in the cardiomyocytes of both EtOH-treated Wt and ALDH2*2 KI mice. Significantly longer APDs were recorded at APD50 as well as at APD90 in cardiomyocytes isolated from EtOH-treated mice as compared to those of their respective normal diet control groups irrespective of genotypes, with the EtOH-treated ALDH2*2 KI cardiomyocytes exhibiting significantly longer APDs than those of the EtOH-treated Wt mice (all *p* < 0.05) (Fig. 6a, b). This indicated that APD prolongation also substantiated our findings pertaining to the morphological optical mapping membrane potential (Fig. 6a). The main depolarization current at the AP upstroke (*V*_max_) of cardiomyocytes, dysfunctional Nav1.5 may act to reduce Na^+^ influx, slow impulse propagation, and harbor conduction heterogeneity^17,18^. In the present study, *V*_max_ in isolated cardiomyocytes of both EtOH-treated mice was substantially diminished compared to those in their respective normal diet control regardless of genotypes (125.9 ± 6.3 vs. 146.9 ± 9.6 9 mV/s for Wt mice, *p* = 0.04; 97.6 ± 8.0 vs. 140.4 ± 13.9 mV/s for ALDH2*2 KI mice, *p* < 0.01), with the EtOH-treated ALDH2*2 cardiomyocytes further exhibiting significantly lower *V*_max_ than that of the EtOH-treated Wt mice (*p* < 0.01, Fig. 6c). There were no significant differences between the resting membrane potential and action potential amplitude of isolated cardiomyocytes of all groups (Fig. 6d).

**Fig. 7:**
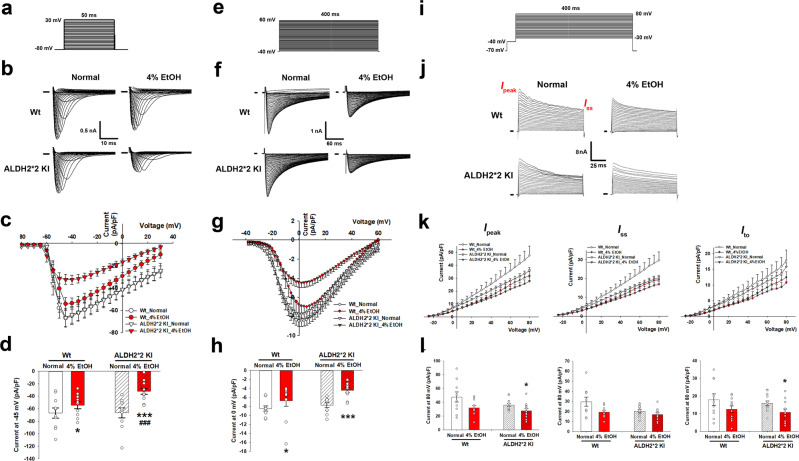
Single cardiomyocyte patch clamp recording of multiple ion current components. *I*_Na_ and *I*_Ca_ were elicited via a 50 (**a**) and 400 ms (**e**) depolarizing step, respectively, with representative traces (**b**, **f** for *I*_Na_ and *I*_Ca_) and current-voltage relationships showing *I*_Na_ and *I*_Ca_ density (**c**, **g**) among different mouse groups. Markedly diminished *I*_Na_ density of cardiomyocytes in both EtOH-treated mouse groups at −45 mV (with the maximal current) compared to their respective normal diet controls irrespective of genotypes and EtOH-treated Wt mice (**d**). At 0 mV, *I*_Ca_ densities of cardiomyocytes in both EtOH-treated mouse groups were significantly lower, especially in ALDH2*2 KI mice, when compared to that in their respective normal diet control groups (**h**). Details about voltage-dependent *I*_Na_/*I*_Ca_ inactivation curves and recovery of *I*_Na_/*I*_Ca_ from inactivation among different mouse groups are further detailed in Supplementary Figs. 3 and 4. The elicited total outward potassium current (*I*_K_) (**i**), representative traces and current-voltage relationships showing transient outward potassium current (*I*_to_) density among different mice groups. The *I*_to_ was defined as the difference between the peak (*I*_peak_) and steady-state current (*I*_ss_) (**j**). *I*_peak_, *I*_ss_, and *I*_to_ (**k**) densities were significantly decreased in cardiomyocytes of both EtOH-treated mice groups. Both *I*_peak_ and *I*_to_ density of cardiomyocytes was significantly lower only in EtOH-treated compared to their respective normal diet control groups of ALDH2*2 KI genotype. These comparisons were not significant in Wt mice groups (**l**). *p < 0.05, **p < 0.01, ***p < 0.001 for any 4% EtOH vs normal diet control within each genotype group (ALDH2*2 KI or Wt); ^#^*p* < 0.05, ^##^*p* < 0.01, ^###^*p* < 0.001 for 4% EtOH ALDH2*2 KI vs 4% EtOH Wt. *n* = 10 independent cells in each group. Error bars represent standard error.

To elucidate the mechanisms underlying the reduction in *V*_max_ action potential in alcohol-treated mouse cardiomyocytes with or without *ALDH2* mutation, we compared the cardiomyocyte sodium current (*I*_Na_) among different groups (Fig. 7a–d). *I*_Na_ was elicited by a 50 ms depolarizing step from −80 mV to +30 mV with an increment of 5 mV from a holding potential of −80 mV. Representative traces and current-voltage relationships showed that *I*_Na_ in the cardiomyocytes isolated from both EtOH-treated mouse groups were markedly diminished, especially in those derived from the EtOH-treated mice compared to that of their respective normal diet control groups (−54.28 ± 5.69 vs −66.87 ± 8.09 pA/pF at −45 mV for Wt mice, *p* < 0.05; −32.23 ± 4.42 vs. −66.50 ± 7.93 pA/pF at −45 mV for ALDH2*2 KI mice, *p* < 0.001) (Fig. 7b–d). Affected *I*_Na_ kinetics in 4% EtOH-treated ALDH2*2 KI mice was analyzed using an *I*_Na_ inactivation curve. Different voltage levels of conditioning pre-pulses (−120 to 30 mV) were applied for 200 ms to induce channel inactivation, in order to examine the inactivation of voltage-dependent *I*_Na_. A second pulse (−30 mV) was then used to depolarize the membrane potential for 50 ms (Supplementary Fig. 7a). The amplitude of *I*_Na_ was then normalized to the maximal current amplitude (Supplementary Fig. 7b), and the traces were fitted using the Boltzmann equation model (Supplementary Fig. 7c). The results showed that the *I*_Na_ inactivation curve in the cardiomyocytes of ALDH2*2 KI mice treated with 4% EtOH was substantially left-shifted with a steeper slope and V_0.5_ observed compared to normal diet ALDH2*2 KI mice or EtOH-treated Wt mice (both *p* < 0.01, respectively), indicating diversely affected *I*_Na_ kinetics. However, EtOH-treated Wt mice only showed a slight yet non-significant shift in the inactivation curve of cardiomyocytes compared to normal diet Wt mice (Supplementary Fig. 7d). On the other hand, recovery of *I*_Na_ from inactivation was determined using a typical paired-pulse protocol. After a −80 mV holding potential, 2 identical −30 mV pulses for 30 ms were separated in each intervals (0–380 ms) (Supplementary Fig. 7e). Currents against intervals between paired pulses were generated and plotted (Supplementary Fig. 7f) and the traces were fitted by a single exponential equation (Supplementary Fig. 7g).

Both EtOH-treated groups showed a significant right-shift with an increased time constant (*τ*) in the recovery curves from inactivation when compared to those of their respective normal diet control groups (*p* = 0.01 and *p* < 0.01 for Wt and ALDH2*2 KI mice groups, respectively), indicating diversely altered AP kinetics (Supplementary Fig. 7h). Overall, EtOH-treated ALDH2*2 KI mice showed more significant cardiomyocyte sodium current (*I*_Na_) reduction and recovery time constant (*τ*) prolongation in the recovery curves from inactivation compared to EtOH-treated Wt mice (Fig. 7b–d; Supplementary Fig. 7). We further investigated the role of calcium currents (*I*_Ca_) in APD and compared cardiomyocyte *I*_Ca_ of different mouse groups (Fig. 7e–h). *I*_Ca_ was elicited by a 400 ms depolarizing pulse from −38 to +60 mV with an increment of 2 mV from a holding potential of −40 mV. *I*_Ca_ density in the cardiomyocytes of both EtOH-treated mice was diminished compared to that of their respective normal diet control (−6.72 ± 1.28 vs −8.48 ± 0.5 pA/pF at 0 mV for Wt mice, *p* < 0.05; −4.41 ± 0.54 vs. −7.75 ± 0.55 pA/pF at 0 mV for ALDH2*2 KI mice, *p* < 0.001) (Fig. 7f–h), again, with the cardiomyocytes isolated from the EtOH-treated ALDH2*2 KI mice showing the most pronounced decrease in their *I*_Ca_ density. The 4% alcohol treatment also shifted the time-dependent recovery curve to the right and slowed down the recovery of *I*_Ca_ from inactivation. To examine the inactivation of voltage-dependent *I*_Ca_, different voltage levels of conditioning pre-pulses (−40 to 10 mV) were applied for 200 ms to induce channel inactivation. A second pulse (0 mV) was then applied for 100 ms to depolarize the membrane potential (Supplementary Fig. 8a). The amplitude of *I*_Ca_ was then normalized to the maximal current amplitude (Supplementary Fig. 8b), and the traces were fitted using the Boltzmann equation model Supplementary Fig. 8c). No significant changes were observed between the *I*_Ca_ inactivation curves of the cardiomyocytes of all mice groups (Supplementary Fig. 8d). On the other hand, recovery of *I*_Ca_ from inactivation was determined using a typical paired-pulse protocol. After holding a potential of –40 mV, two identical 200 ms long 0 mV pulses were applied with intervals of 0–85 ms in between (Supplementary Fig. 8e). Currents against intervals between paired-pulses were plotted (Supplementary Fig. 8f), and the traces were fitted via a single exponential equation (Supplementary Fig. 8g). The time constant (*τ*) was significantly increased in the cardiomyocytes from both EtOH-treated mice when compared to that of their respective normal diet control groups regardless of genotypes (*τ*; both *p* < 0.01; Supplementary Fig. 8h), indicating that repolarizing *I*_*Ca*_ kinetics was significantly perturbed due to alcohol treatment.

To clarify the association between the transient outward potassium current (*I*_to_) and APD prolongation in cardiomyocytes of alcohol-treated Wt and ALDH2*2 KI mice, we compared the *I*_to_ of cardiomyocytes from different groups in the voltage-clamp mode (Fig. 7i–l). The total outward potassium current (*I*_K_) was elicited via a 5 ms depolarization to −40 mV, followed by a 400 ms depolarizing pulse from −30 mV to +80 mV, with an increment of 5 mV from a holding potential of −70 mV. *I*_to_ was defined as the difference between the peak current (*I*_peak_) and the steady-state current (*I*_ss_) (Fig. 7j). The current-voltage relationships showed that *I*_peak_, *I*_ss_, and *I*_to_ in the cardiomyocytes of both EtOH-treated Wt and ALDH2*2 KI mice were significantly lower compared to normal diet Wt mice (Fig. 7k). At +80 mV, both *I*_peak_ and *I*_to_ densities of cardiomyocytes of the EtOH-treated mice were significantly lower compared to those of their normal diet control groups of ALDH2*2 KI genotype (23% and 32% reduction, respectively) though not significantly different between EtOH-treated and normal diet mice groups of Wt genotype (*I*_peak_ and *I*_to_: 27.68 ± 3.18 vs. 35.87 ± 2.26 pA/pF and 10.77 ± 1.77 vs. 15.83 ± 1.33 pA/pF for EtOH-treated KI vs. normal diet KI, both *p* < 0.05; 31.79 ± 3.11 vs. 47.52 ± 7.25 pA/pF and 12.45 ± 1.81 vs. 17.87 ± 3.33 pA/pF for EtOH-treated Wt vs normal diet Wt, *p* = 0.08 and 0.15, respectively; Fig. 7l). The *I*_ss_ density of cardiomyocytes from both EtOH-treated mice were comparable to that of their respective normal diet control irrespective of genotypes (Fig. 7l). Neither *I*_to_ inactivation (Supplementary Fig. 9a–d) nor recovery (Supplementary Fig. 9e-h) of *I*_to_ of the cardiomyocytes was found to differ among different groups. The inward rectifier potassium current (*I*_K1_) was comparable among all four groups, supporting the finding that RMP was similar across all groups (Supplementary Fig. 10). Taken collectively, our optical mapping and simulation mimicking human VA, as well as the results of patch clamp measurements of single-cell action potentials indicated that the ALDH2*2 KI genotype was the characteristic component associated with VA in humans engaged in light-to-moderate alcohol consumption.

## Discussion

It has been proposed that the threshold for the toxic effect of alcohol is significantly lower in human subjects carrying the ALDH2*2 variant^19^. A reduction in the safety threshold of alcohol exerts a health impact, involving clinical implications for an estimated 540 million population comprising nearly 35%–45% of the global East Asian population^10,12–14^. Here, we report that the QTc durations (*ALDH2* rs671 G/A or A/A) were significantly prolonged in human subjects who are light-to-moderate alcohol users (median dose: 12.7 [IQR: 6.3~32.8] g/day, or median standard drink: 0.9 [IQR: 0.5~2.3] drinks/day; 1 U.S. standard drink = 14 g of pure alcohol) carrying *ALDH2* variant genotypes, compared to those habitual alcohol users carrying the *ALDH2* wild genotype and alcohol non-users^3,4^. Using ALDH2*2 KI mice that were administered a 4% alcohol diet for 7 weeks, we recapitulated the human electrocardiographic phenotype of QT prolongation with VA vulnerability. VA vulnerability was exclusively observed in live alcohol-fed ALDH2*2 KI mice and provoked in ex vivo Langendorff-perfused hearts derived from alcohol-fed ALDH2*2 KI mice via programmed electrical stimulation. Induction of VA in ALDH2*2 KI mice fed a low alcohol dose appeared to hinge on pathologically elicited myocardial collagen deposition along with distinctive Cx43 and sarcolemmal Nav1.5 remodeling affecting depolarization. Impaired repolarizing ionic mechanisms (e.g., *I*_to_ density reduction) observed from single-cell patch-clamped cardiomyocytes and abnormal CaMKII signaling further aggravated prolonged APD with increased VA susceptibility in alcohol-fed ALDH2*2 KI mice.

Acute and heavy alcohol exposure has been shown to trigger several pathological, signaling cascades leading to arrhythmias^5,20,21^. Although chronic heavy drinking has been shown to cause life-threatening arrhythmias, it remains unclear whether ALDH2 enzyme deficiency contributes to unfavorable electrophysiological features at a lower safe threshold compared to those without ALDH2 deficiency. Based on a 5-year observation period which was conducted as a part of the present study, we found that the threshold level relating daily ethanol exposure to the risk of developing clinical VAs was below 1.0 standard drink per day (9.6 g/day or 0.7 drinks/day) for humans carrying *ALDH2* variants as compared to normal *ALDH2* human subjects (14.1 g/day or 1 drink/day). This finding suggests that inactive ALDH2 enzyme carriers consuming light-to-moderate levels of alcoholic beverages may face potentially life-threatening VA events. Additionally, individuals carrying the inactive form of *ALDH2* were more likely to experience extremely unfavorable outcomes due to VA susceptibility. In accordance with these findings, the current study indicated that high-risk VA episodes along with rotors formation by optical mapping (Fig. 5h, i) were only inducible in ALDH2*2 KI mice treated with a diet incorporating 4% EtOH, a dose which is slightly lower than a diet incorporating the accepted moderate alcohol dose of 5% EtOH, reported by another study^22^. Compared to Wt mice fed a 6% EtOH diet continuously for 14 weeks, which demonstrated prominent electrophysiological disturbances in a previous study of ours, Wt mice fed a 4% EtOH diet for continuous 14 weeks showed only modest ventricular electrophysiological effects without provocable VA^16^. Notably, the BAC of 40–50 mg/dL (8–12 mM) in our 4% EtOH-treated Wt mice corresponded to the modest dose in humans^23^ and the light-to-moderate alcohol exposure in mouse C57BL/6 experimental models^24^. This alcohol concentration was also considered to be a low dose (2–20 mM) with respect to alcohol-related central nervous effects^23^. In another study, the acute effect of APD prolongation in human ventricular cardiomyocyte using a simulation model was only observed at a high ethanol concentration of 80 mM^25^, whereas APD prolongation in ALDH2*2-derived mouse cardiomyocyte was observed at a lower threshold of 12 mM ethanol.

The findings of the current study indicated that Cx43 was upregulated in 4% EtOH-treated Wt mice for 7 weeks. However, we observed that Cx43 was significantly downregulated in Wt mice with 4% EtOH treatment for continuous 14 weeks in our previous work^16^ and in 4% EtOH-treated ALDH2*2 KI mice for only 7 weeks in the present study, which perhaps indicated that ALDH2 deficiency would accelerate Cx43 abnormality with same dose of ethanol exposure. As reported in a previous study of ours, Cx43 was upregulated in wild-type C57BL/6 J mice given 36% alcohol as the only source of drinkable fluid^26^. Nevertheless, both EtOH-treated Wt and ALDH2*2 KI mouse groups displayed enhanced Cx43 lateralization (Fig. 3c), which likely promoted non-junctional hemichannel formation and facilitated current leakage with enhanced Ca^2+^ dynamics^27,28^. These arrhythmogenic Cx43 remodeling features combined with elicited interstitial fibrosis, further impaired cell-to-cell coupling and partly contributed to dispersed impulse propagation leading to slowed CV and promoted re-entry with pro-arrhythmic effects^17,29,30^. Modest Cx43 remodeling in conjunction with factors influencing electrical conduction (e.g., reduced *V*_max_) can also be arrhythmogenic. Furthermore, downregulation of Nav1.5 (or *I*_Na_) with a slower phase 0 may also contribute to APD prolongation^17,18^. Therefore, we further examined *I*_Na_ in EtOH-treated ALDH2*2 KI mice using isolated single-cell cardiomyocyte patch-clamping^29^. A previous study of ours has shown that administering 4% EtOH for 14 weeks exerted modest effects, such as sarcolemmal Nav1.5 reduction (~30%) and dysfunction, on C57BL/6 WT mice^16^. In comparison, the reduction of membranous Nav1.5 and *I*_Na_ were more pronounced in ALDH2*2 KI mice compared to EtOH-treated Wt mice treated with 4% EtOH for 7 weeks, though both EtOH-treated Wt and ALDH2*2 KI mice showed diminished *V*_max_ and impaired Na^+^ influx with shared abnormal gating properties compared to their respective normal diet controls in our patch-clamp study. Furthermore, as both the resting membrane potential and *I*_K1_ may influence *V*_max_ and *I*_Na_, comparable values of these tests (Fig. 6d & Supplementary Fig. 10) indicated that the observed reduction in *V*_max_ and diminished *I*_Na_ may be attributable to excessive downregulation of Nav1.5 (as observed in EtOH-treated ALDH2*2 KI mice) or to inner Nav1.5 dysfunction. Overall, these findings also suggest that dysregulated Cx43 and Nav1.5 in EtOH-treated ALDH2*2 KI mice led to a more profound loss of conduction reserve during depolarization, thereby providing electrophysiological substrates that favor the initiation of re-entrant circuit formation and VA episodes.

Kv ion channel proteins, especially *I*_to_, may play a pivotal role in determining cardiomyocyte APD, since the inward rectifier potassium current (*I*_K1_) was comparable among different mouse groups in our study^29,31^. Notably, both Kv1.4 and Kv4.2 play essential roles as ion channels responsible for ventricular repolarization in mice with Kv4.2 serves as the dominant ventricular Kv ion channel protein^32–34^. Therefore, downregulated Kv4.2 expression further led to a decline in *I*_to,f_, and a prolongation of APD^35^. In the present study, both the sarcolemmal Kv1.4/Kv4.2 were markedly reduced in EtOH-treated ALDH2*2 KI mice compared to those of their respective EtOH-treated Wt mice, indicating a critical dysfunction in *I*_to_ and repolarization processes from ALDH2 enzyme deficiency exposed to EtOH that may facilitate arrhythmia substrate formation. By contrast, Kv4.3, a minor functioning I_to,f_ channel in mice appeared to have been distinctly regulated in our EtOH-treated ALDH2*2 KI mice to compensate for the suppressed Kv4.2 or the diminished *I*_to_, as reported in other studies^33,36^. Upregulated Kv4.3 is also known to counteract the consequences of electrical dispersion-induced fatal VAs from hyper-activated CaMKII and downstream signaling with an attempt to reverse ventricular electrical/structural remodeling in certain pathological heart conditions^37–39^. Nevertheless, defective Ca^2+^ homeostasis due to CaMKII overexpression caused by alcohol treatment in ALDH2*2 KI mice likely activates CaMKII-dependent downstream signaling (e.g., sodium-calcium exchanger activation)^40^ by generating an inward, depolarizing current that further contributes to AP prolongation^41,42^. In agreement with previous findings which indicate that accumulated acetaldehyde may inhibit *I*_Ca,_ through multiple mechanisms, for example, disturbed intracellular Ca^2+^ homeostasis by directly favoring RyR2-mediated Ca^2+^-leak and RyR2 inhibition, disturbed Ca^2+^ phosphorylation, or SERCA-mediated Ca^2+^-leak from CAMKII activation, thus limiting the amount of Ca^2+^ to be released from the sarcoplasmic reticulum upon stimulation. We, therefore, speculated that such effects may also be possible and aggravated under ALDH2*2 KI conditions^21,43–45^. The slower and right-shifted *I*_Ca_ activation curve (Fig. 7g), in conjunction with a slower recovery time constant (τ) from inactivation (Supplementary Fig. 8), may predispose the cardiomyocytes to AP prolongation thereby increasing the propensity for arrhythmias. Considered together, these features likely act simultaneously and synergistically to promote repolarizing arrhythmogenic substrate and rotors formation with higher VAs vulnerability during programmed stimulation in ALDH2*2 KI mice treated with 4% EtOH^46^. Future studies are needed to demonstrate whether these mechanisms and electrical perturbations are the causes underlying increased susceptibility of ALDH2*2 carrying human subjects who engage in chronic light-to-moderate alcohol use, to arrhythmias.

**Fig. 8:**
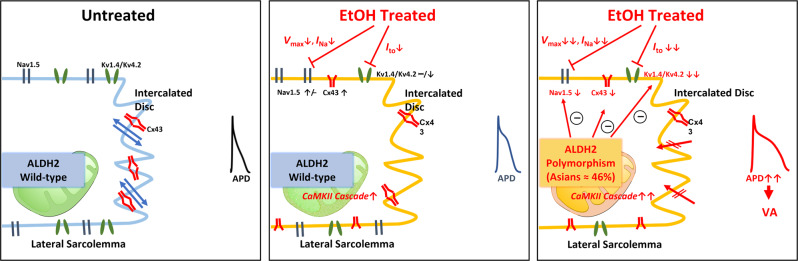
Schematic diagram illustrating the effect of ALDH2 knock-in (KI) on cardiac electrophysiological properties with and without alcohol treatment. Changes of cardiac electrophysiological properties in conditions of (1) Wild-type (Wt) without EtOH use (left panel), (2) Wt with EtOH use (middle panel), and (3) ALDH2 variant (polymorphism) with EtOH use (right panel).

In summary, our findings address the current knowledge gap pertaining to the alcohol safety thresholds needed to prevent VAs in human subjects carrying Wt *ALDH2* and those carrying the common alcohol intolerant ALDH2*2 inactive variant. To the best of our knowledge, this is also the first study to provide comprehensive data related to the ventricular electrophysiological properties of Wt *ALDH2* and ALDH2*2 variant carriers who are chronically exposed to alcohol levels ranging from light-to-modest. We demonstrated that phenotypic APD prolongation linked to VAs in human subjects known for carrying the ALDH2*2 variant as well as light-to-modest alcohol intake, fully substantiated the findings of our animal studies using ALDH2*2 KI mice treated with a corresponding low dose of alcohol. Mechanistic studies have shown ventricular re-entry substrates with rotor formation via optical mapping. At the molecular level, a wide range of ion channels and connexin remodeling was found to drive dysfunctional depolarization/repolarization currents, leading to increased susceptibility to VAs (Fig. 8). These findings indicate that the ALDH2*2 genotype is the susceptible component of VA in humans with light-to-moderate alcohol consumption and underscore the risk of lethal VAs induced by low-to-moderate alcohol use. Interpretation of current study findings should be cautious as our experimental mice model was treated with a 4% alcohol diet as the only source of food, which may vary from the pattern and frequency of alcohol use in humans. Since ALDH2*2 affects a large global population consisting of 540 million East Asians, many of whom are habitual light-to-moderate alcohol drinkers, our findings may warrant confirmation via further large-scale epidemiological studies in the future. Asian *ALDH2* missense variants, other than the ALDH2*2 variant, have recently been compiled by the Human Genome Aggregation Database (gnomAD, https://gnomad.broadinstitute.org/)^47^. Several of these *ALDH2* variants have displayed reduced alcohol-metabolizing activity in vitro^47^. It is probable that carriers of such *ALDH2* variants in other ethnic groups may also become vulnerable to VA events via the use of light-to-moderate alcohol. Likewise, our recent work also showed *ALDH2* variants are prone to the development of atrial fibrillation and impaired atrial function with light-to-moderate alcohol use^48^. Hence, there is a growing need for public health policy makers to evaluate and formulate safe alcohol drinking guidelines for a large segment of the affected ALDH2-deficient population. Small-molecule enzyme activators of ALDH2*2, such as Alda-1, have been discovered^11^. From a preventive standpoint, such compounds may serve as protective agents for subjects carrying the ALDH2*2 variant, who are frequent alcohol users or are addicted to alcohol.

## Methods

### Selection and long-term monitoring of light-to-moderate alcohol consumption and QTc phenotypes in human subjects

We prospectively recruited 268 study participants from our outpatient clinics via a structured questionnaire concerning habitual alcohol use. ALDH2 SNP rs671 genotyping was conducted in 260 study participants, of which 256 completed clinical demographic information, following selection based on exclusion criteria (Supplementary Fig. 1a). We gathered 12-lead body surface ECG results, anthropometrics (such as body height, weight, and waist circumferences), biochemical information (including lipid profiles and renal function defined as eGFR using the MDRD formula), and background medical histories, including the presence of hypertension, type 2 diabetes, hyperlipidemia treatment, CAD, and associated medications used by all participants. All our study participants were heart failure-free and had no urgent medical conditions. Informed consent was obtained from all study participants. These participants were followed up for at least five years regarding alcohol use. Our questionnaires and surveys indicated that their alcohol consumption pattern was stable for at least 5 years. Daily alcohol intake dose was estimated using a structured questionnaire in which different types of alcoholic beverages consumed as well as amounts and frequency of use were self-reported^49^. In brief, study participants were asked to indicate how often they consumed beer, liquor, wine, and strong wine, as well as the amount consumed on each single occasion. Weekly alcohol consumption for each participant was calculated in grams of pure alcohol by multiplying the product of the frequency of each consumed alcoholic beverage and the exact dose of ethanol (derived from the amount and ethanol concentration [i.e., 3.5–4.5% for beer], by the specific gravity of ethanol (0.79 g/ml) in each kind of alcohol beverage) as follows:

1 $${{{{{\rm{Exact}}}}}}\,{{{{{\rm{ethanol}}}}}}\,{{{{{\rm{dosage}}}}}}= \,\, 	{{{{{\rm{quantity}}}}}}\,{{{{{\rm{of}}}}}}\,{{{{{\rm{alcohol}}}}}}\,{{{{{\rm{beverage}}}}}}\,{{{{{\rm{(mL)}}}}}}\\ 	\times {{{{{\rm{alcohol}}}}}}\,{{{{{\rm{concentration}}}}}}\,{{{{{\rm{in}}}}}}\,{{{{{\rm{that}}}}}}\,{{{{{\rm{beverage}}}}}}\\ 	\times {{{{{\rm{specific}}}}}}\,{{{{{\rm{gravity}}}}}}\,{{{{{\rm{of}}}}}}\,{{{{{\rm{alcohol}}}}}}.$$

Study participants were categorized as alcohol non-users (defined as those who did not use alcoholic beverages or those who consumed <14 g of alcohol (equal to 1 standard drink) on a weekly basis) and habitual alcohol users (those who consumed >1 standard drink/week). In order to focus on human subjects with only light-to-moderate alcohol consumption, 3 (who were classified as heavy alcohol drinkers (alcohol use ≥90 g/day or 6.4 drinks/day) of the initial 256 study participants were excluded. This resulted in a total of 253 study participants, including 196 habitual light-to-moderate alcohol usttracks, entering our final analysis and follow-up (Fig. S1A). These 196 habitual light-to-moderate alcohol users and 57 alcohol non-users were further categorized into two groups according to their *ALDH2* genotypes as follows: ALDH2*1 wild-type (Wt); and *ALDH2* variant (ALDH2 GA or AA genotype) (*ALDH2* Vt) (Table 1). This study passed the Institutional Review Board of MacKay Memorial Hospital (14MMHIS069) and complied with the Declaration of Helsinki. The datasets generated and/or analyzed during the current study are available from the corresponding author upon reasonable request.

### Body surface electrocardiogram (ECG) parameters in human

Each study participant received a standardized 12-lead body surface ECG recording (Page Writer Trim III; Philips, Andover, MA, USA). Parameters pertaining to PR interval, QRS duration, and QT intervals (with and without correction to RR interval: QTc) were obtained and automatically analyzed via software with the paper speed set at 50 mm/s, which enabled precise and reliable QRSd and QT index measures.

Baseline and serial ECGs were performed annually or followed twice per year in outpatient clinics for up to 5 years. Furthermore, Holter monitoring (for continuous 24 hours) was arranged based on the clinical judgment of cardiologists and the clinical symptoms (i.e., palpitation, dizziness, or chest discomfort) or signs (e.g., suspected arrhythmias) of study participants. Clinical VAs was defined as >1 premature ventricular contraction on a single 10-second ECG recording or >30 premature ventricular contractions in an hour (averaged by 24-hour recording) at baseline or during follow-up (up to 5 years), which is reportedly associated with a higher rate of sudden cardiac death^50^.

### Effect of alcohol on cardiac electrophysiology using an animal model of ALDH2*2 KI mice

The ALDH2*2 KI animal study complied with the institutional and national guidelines for the care and use of laboratory animals (Taiwan Animal Protection Law; Scientific Application of Animals, 1998). All animals used in this study (homozygous ALDH2*2 mice and their wild-type littermates) had a C57BL/6 genetic background, and the protocols were reviewed by the institutional board (MMH-A-S-107-69). Four-month-old wild-type male C57BL/6 mice or homozygous ALDH2*2 KI mice were assigned into two diet groups^16^. These were, (i) the normal diet group (ad libitum liquid diet; dry mix #F1259SP Bio-Serv^®^ Advancing Science. Enriching Animals, USA Test with warm tap water); and (ii) the 4% EtOH group (4% v/v alcohol liquid diet; dry mix #F1697SP, Bio-Serv^®^ supplemented with maltose-dextrin, ethanol, and warm tap water). Liquid diet remained the only source of fluid and food the mice had access to for 7 continuous weeks. In total, our study design consisted of four mouse groups: (i) Wt/normal diet, (ii) Wt/4% EtOH, (iii) ALDH2*2 KI/normal diet, and (iv) ALDH2*2 KI/4% EtOH. In the normal diet group, maltose-dextrin was substituted iso-calorically with ethanol. One hour after feeding, all mice were euthanized under deep anesthesia, and venous blood drawn and collected from the tail (0.1–0.2 ml). Serum samples were collected and centrifuged, and the plasma was stored at −80 °C. BACs were determined using an EnzyChrom™ Ethanol Assay Kit (ECET-100, BioAssay Systems). The experimental protocol was approved by the Animal Care and Use Committee from the Institutional Review Board of MacKay Memorial Hospital. The mice were euthanized by isoflurane anesthesia with transcardial perfusion with saline. A schematic of the mouse study is shown (Fig. 1).

### Body surface ECG parameters and monitoring for mice

Body surface ECG was performed under anesthesia using 1–2% isoflurane (FORANE, ABBOTT Laboratories Ltd., England) in a combination of oxygen and nitrous oxide mixed with air at a ratio of 50:50. Upon stabilization, our experimental mice were subjected to detailed body surface ECG examinations (at a heart rate of ~450–550 beats/min) with electrodes connected to a Biopac monitoring system (Biopac Systems, Inc., Goleta, CA, USA). The body surface ECG was conducted using ECG electrodes inserted subcutaneously in all four limbs, and sequential ECG recordings were acquired at a 2 kHz sweep-speed for 10 min. ECG results for mice were stored as raw data and recorded at baseline (16 weeks old) and at the study endpoint (23 weeks old) before sacrifice. Data were stored for offline analysis using custom 2 MATLAB scripts analyzed using commercialized software (Biopac Student Lab Analysis 4.1.0) covering 30–35 heartbeats. The PR interval was measured from the beginning of the P wave to the peak of the R wave. The QRS interval was measured from the beginning of the Q wave until the point at which the S wave crossed the baseline point. The QT interval was measured from the beginning of the Q wave until the point at which the T wave declined to 90% (T90) from the peak. Adaptive heart rate-corrected QT values were derived using a modification of Mitchell’s formula for murine ECG as:

2 $$	{{{{{\rm{QTc}}}}}}={{{{{\rm{QT}}}}}}/{{{{{\rm{square}}}}}}\,{{{{{\rm{root}}}}}}\,{{{{{\rm{of}}}}}}({{{{{\rm{RR}}}}}}/100).\\ 	{{{{{\rm{Quantification}}}}}}\,{{{{{\rm{of}}}}}}\,{{{{{\rm{these}}}}}}\,{{{{{\rm{measures}}}}}}\,{{{{{\rm{were}}}}}}\,{{{{{\rm{blinded}}}}}}\,{{{{{\rm{to}}}}}}\,{{{{{\rm{group}}}}}}\,{{{{{\rm{assignment}}}}}}.$$

### Immuno-confocal microscopy for Cx43 and immunofluorescence microscopy for ion channel proteins

In order to analyze myocardial Cx43 morphology and remodeling patterns, immunolabeled Cx43 from myocardial samples was imaged and examined via confocal laser scanning microscopy using a Leica TCS SP equipped with an argon/krypton laser. Morphological and structural remodeling of various ion channel proteins including Nav1.5, Kv1.4, Kv4.2, Kv4.3, Cav1.2, and Cav1.3 were examined via immunofluorescence microscopy. Mouse hearts were first perfused with normal saline. All ventricular samples were subsequently embedded in OCT solution and stored at −80 °C. Frozen tissue was sliced into 3 µm sections and fixed with 4% paraformaldehyde, permeabilized with 0.5% Triton X-100 for 30 min, and then blocked in blocking solution (0.1% bovine serum albumin and 0.1% Triton X-100 in Dulbecco’s phosphate-buffered saline) for an hour. Double staining of wheat germ agglutinin and connexin 43 were also performed for cardiac remodeling detection. FITC-conjugated WGA (Vector, Burlingame, CA, USA) was used to detect cell borders. Anti- Cx43 antibody (Sigma-Aldrich, St. Louis, USA) was used to detect Cx43. Donkey anti-rabbit Cy3 (Chemicon, California, USA) was used to visualize immunolabeled connexin43 (Cx43).

For ion channel and WGA double labeling, Nav1.5 (1:200, rabbit polyclonal, Alomone Labs), Cav1.2 (1:100, rabbit polyclonal, Alomone Labs), Cav1.3 (1:100, rabbit polyclonal, Alomone Labs), Kv1.4 (1:100, rabbit polyclone, Alomone Labs), Kv4.2 (1:100, rabbit polyclone, Alomone Labs), and Kv4.3 (1:100, rabbit polyclone, Alomone Labs) antibodies were incubated overnight at 4 °C, followed by labeling with goat-anti-rabbit Alexa 488 (1:400, Invitrogen). And in the next day, WGA conjugates with Alexa 594 (1:500, Invitrogen) were incubated for 2 hours at 25 °C. All sections were mounted with an anti-fade containing DAPI.

Images were collected using a 40× objective lens, and a zoom 1.0 for Cx4.3/WGA staining; zoom 4.0 for ion channel/WGA staining of computer setting. Each recorded image consisted of 1024 × 1024 pixels. Projection views of four consecutive optical sections were taken at 0.25 or 1 μm intervals in the middle of the sections and recorded for analysis. All images were processed with ImageJ or Adobe Photoshop CS6 software.

Image analysis of Cx43 was conducted using QWIN image analysis software (Leica) for analysis of myocardial Cx43 distribution, and the following information and assessments were obtained from each mouse group including (i) the total area of immunolabeled Cx43 (per image slice), (ii) the total area of immunolabeled Cx43 per cardiomyocyte area, and (iii) the percentage of immunolabeled Cx43 area along the lateral borders divided by the total area of immunolabeled Cx43 proteins in individual cardiomyocytes.

### Western blotting for Cx43, ion channel proteins, and calcium/calmodulin-dependent protein kinase

Western blotting of whole-tissue preparations from myocardial tissue samples for Cx43 (including total, phosphorylated [functional] [p-Cx43], and non-phosphorylated [np-Cx43] isoforms of Cx43), calcium/calmodulin-dependent protein kinase II (CaMKII, both total and oxidized [ox-CaMKII] forms), ion channels, and relevant ion transporter proteins responsible for action potential generation was conducted using appropriate antibodies. Cytoplasmic and membranous fractions of key ion channels or proteins that are associated with electrophysiological properties were further tested. Tissue samples of left ventricular free wall (as whole-tissue preparations) from liquid nitrogen were lysed using 1% NP40 cell lysis buffer, containing a phosphatase inhibitor cocktail tablet (Roche) and a protease inhibitor cocktail tablet (Roche), and homogenized via sonication. Cytoplasmic and membrane proteins of the left ventricular free wall were prepared using a CNM compartment Protein Extraction Kit (K3012010, BioChain, USA). Total protein was estimated using Lowry’s method (DC Protein Assay Kit, Bio-Rad, USA).

SDS-polyacrylamide gel electrophoresis was performed using mini-gels made of 5% stacking gels and 10–12% separation gels. Next, 30–40 µg of each tissue protein, 100 µg of cytoplasm fraction, or 50 µg of membrane fraction, were mixed with sample buffer (2.5% 2-mercaptoethanol and 1% bromophenol blue) to produce a final volume of 20–30 µL, loaded in each lane, subjected to electrophoresis (80 V for running in stacking gel and 120 V in separation gel; constant voltage) and transferred (80 V, constant voltage for 150 min on ice). PVDF membrane (Perkin Elmer, USA) was detected using primary antibodies specific for Collagen-1 (1:1000, mouse monoclonal, Sigma), Cx43 (1:250, mouse monoclone, BD Biosciences), Cx43 (1:1000, rabbit polyclone, Sigma-Aldrich) to recognize the phosphorylated (functional) (p-Cx43) form and non-phosphorylated (np-Cx43 [Cx43-P0]) isoforms of Cx43. TGF-beta 1 (1:500, mouse monoclone, Santa Cruz), Nav1.5 (1:200, rabbit polyclonal clone, Alomone Labs), Kv1.4 (1:200, rabbit polyclonal clone, Alomone Labs), Kv4.2 (1:200, rabbit polyclone, Alomone Labs), Kv4.3 (1:200, rabbit polyclone, Alomone labs), Cav1.2 (1:250, rabbit polyclone, Alomone Labs), Cav1.3 (1:250, mouse monoclone, Gene Tex), NCX1 (1:1000, mouse monoclone, Gene Tex), p (Thr286)- Calcium/calmodulin-dependent protein kinase II (CaMKII) (1:2000, mouse monoclonal, Gene Tex), ox-CaMKII (1:1000, rabbit polyclonal clone, Gene Tex), and total CaMKII (1:1000, rabbit polyclonal clone, Gene Tex) at 4°C overnight. The blots were further incubated with secondary antibodies for 1 h at room temperature. Immunoreactivity was visualized using an ECL solution (Perkin Elmer, USA) to develop the images. To normalize expression levels, blots were stripped and incubated with anti-GAPDH antibody (1:50,000, mouse monoclonal, Sigma-Aldrich), which was used as an internal control. To normalize membrane protein expression levels, we used membrane marker protein Na^+^ K^+^- ATPase (1:1000, Rabbit polyclonal, Cell Signaling) as an internal control. For western blotting, quantitative densitometric scanning and analysis were performed on the blots using the MultiGel-21 image system (TOPBIO CO. Taiwan) to develop images and the Gel-Pro software for analysis. Uncropped original versions of these analysis were available in Supplementary Figs. 11–15.

### Ex vivo optical mapping of VA in Langendorff-perfused mouse hearts

The mice were euthanized under anesthesia for ex vivo mapping of VA. Hearts were immediately excised via thoracotomy and Langendorff-perfused with warm oxygenated Tyrode’s solution (pH 7.4; 95% O_2_, 5% CO_2_, 36–38 °C). One Ag/AgCl electrode was placed on the LV near the apex, while another was attached to the surface of the right ventricle to record the ECG. The isolated mouse heart was stained with a voltage-sensitive dye (Di-4-ANEPPS, 100 μmol/L, ThermoFisher Scientific, Waltham, MA, USA) to record optical membrane potentials following 10 min of stabilization. Blebbistatin (5–10 μmol, TOCRIS, Bristol, UK) was directly added to Tyrode’s solution to reduce motion artifacts. Hearts were uniformly illuminated with a green light-emitting diode at a wavelength of 505 ± 20 nm to activate Di-4-ANEPPS. Fluorescence images were captured through a 600-nm long-pass filter using a CMOS camera (1000 frames/second, SciMedia, USA). Vulnerability to pacing-induced VA was assessed via programmed electrical stimulation through ten cycles of burst pacing (20 Hz, 10 s) which was twice the excitation threshold of the left ventricle for each mouse. The dominant frequency of optical signals during induced VA at each location of the mapped area was recorded and calculated using fast Fourier transform. A dominant-frequency map was constructed using the dominant frequency of all pixels.

### Single cardiomyocyte patch clamp study

The whole-cell patch clamp technique was used to record membrane potential and ionic currents with an Axon CNS 700 B amplifier (Molecular Devices, CA, USA), Digidata 1550 A data acquisition system, and pClamp software (Version 10, Molecular Devices) in current- and voltage-clamp modes. Left ventricular myocytes were enzymatically isolated using the Langendorff heart perfusion technique. Mouse hearts were retrogradely perfused with Krebs buffer (120 mM NaCl, 12 mM glucose, 25 NaHCO_3_, 1.2 KH_2_PO_4_, 1.2 MgSO_4_, and 5.4 KCl. After equilibration, 0.4 mg/mL collagenase (type II, Worthington) in Krebs buffer was perfused for 20 min. Mouse hearts were cut into small pieces and filtered in Dulbecco’s modified Eagle’s medium (Invitrogen, Gibco).

Quiescent cells were placed in a chamber mounted on the stage of an inverted microscope (Eclipse Ti-U, Nikon Corporation, Japan) in a bath solution containing 137, 5.4, 1.8, 1.1, 6, 22, and 0.33 mM of NaCl, KCl, CaCl_2_, MgCl_2_, HEPES, glucose, and NaH_2_PO_4,_ respectively. The pH was adjusted to 7.4 using NaOH. For experiments aimed at detecting *I*_Na_, N-methyl-D-glucamine (91 mM) was used to replace equal concentrations of NaCl. In specific experiments, Cs^+^ (2 mM) and Co^2+^ (1 mM) were added to block potassium and calcium currents, respectively. Heat-polished glass electrodes (tip resistances of approximately 2 MΩ when filled with an internal solution through a pipette) were prepared from borosilicate glass capillaries (outer diameter 1.5 mm) using a glass microelectrode puller (PC-10, Narishige International Inc., East Meadow, NY, USA). The internal solution contained 120 mM KCl, 5 mM MgCl_2_, 5 mM MgATP, 10 mM HEPES, and 15 mM EGTA; pH was adjusted to 7.2 using KOH at room temperature. For the *I*_Na_ and *I*_Ca_ measurements, Cs^+^ and tetraethylammonium (TEA) were added to the pipette solution.

### *ALDH2* genotyping for human and mouse samples

TaqMan SNP Genotyping Assays (Applied Biosystems, Foster City, CA, USA) were used to conduct *ALDH2* genotyping of all human participants while direct PCR DNA sequencing was used to determine the genotypes of our experimental mice in our study. In human genotypes, both the *ALDH2* rs671 GA and AA genotypes were defined as carriers of the *ALDH2* variant (*ALDH2* Vt). The absence of the *ALDH2* rs671 variant A allele or GG genotype was defined as an *ALDH2* wild-type (*ALDH2* Wt) carrier. Genomic DNA was extracted from EDTA-containing whole blood samples obtained from each participant using a semi-automated extraction system (Smart LabAssist, Taiwan Advanced Nanotech Inc., Tau-Yuan County, Taiwan) equipped with a TANBead Blood DNA plate (Taiwan Advanced Nanotech Inc.). The DNA samples were then genotyped for target polymorphism, *ALDH2* Glu487Lys (rs671, G/A) of each candidate gene using an established commercial service (Genomics BioSci & Tech. Co., Ltd, Taiwan). The designated SNPs were determined via real-time PCR-based allelic discrimination using reagents for TaqMan SNP Genotyping Assays (Applied Biosystems, Foster City, CA, USA) following the manufacturer’s instructions. TaqMan probes were used to examine *ALDH2* Glu487Lys (rs671, G/A) in the current study. PCR amplification and allelic discrimination were performed using a ViiA7 Real-Time PCR System (Applied Biosystems). The quantitative PCR setting was programmed at 60°C for 30 s with initial denaturation for 10 min at 95 °C, followed by 40 cycles of denaturation at 95 °C for another 15 s and annealing at 60 °C for 1 min. Allele detection and allelic discrimination were performed for 1 min at 60 °C. Raw data were then analyzed using the ViiA7 Software (v1.2.4). Genotyping success rate for the analysis of the target SNP (*ALDH2* Glu487Lys [rs671, G/A]) was >96.6% with a mismatch rate of 0.0%. For the genotyping for *ALDH2* in Mice, mouse DNA was extracted from mouse tail samples using a KAPA Mouse Genotyping Kit (KAPA Biosystems). Tail tip no longer than 1–2 mm was digested in a 100 μl volume containing 88 μl PCR-grade water, 2 μl 10X KAPA Express Extract buffer, and 2 μl KAPA Express Extract Enzyme, at 75 °C for 10 min, followed by 95 °C for 5 min to inactivate the enzyme. PCR was performed with 1 μl DNA in 1× KAPA2G Fast Genotyping Mix and 0.5 μM primers: 5 min denaturation at 95 °C, 35 cycles (20 s at 95 °C, 20 s at 60 °C, 30 s at 72 °C), and final extension at 72 °C for 5 min. The *ALDH2* primers EG534 (GTTCTCTCCGATGACAGGATCAACTGCTAC) and EG536 (CAGACATTAACACACTGGGCATTTAGGTC). PCR fragments amplified by EG534 primers were sequenced directly using EG535 (TACGTTCCCGTGGGCAGAACTGGTGCCTT)^51^.

### Statistics and reproducibility

Human data were analyzed using the STATA 14.0 software package (Stata Corp., College Station, Texas, USA). Continuous data are presented as mean ± standard deviation (SD) and compared by Student’s *t* tests unless specified otherwise. Categorical values, expressed as numbers and percentages, were compared using the chi-square or Fisher’s exact test as appropriate. The association of *ALDH2* polymorphism (as *ALDH2* Vt) with clinical VAs was assessed using logistic regression. We further tested whether the presence of *ALDH2* Vt modifies the association between alcohol intake and VAs or QTc prolongation. Animal data were subjected to unpaired Student’s *t* tests or Mann–Whitney *U* tests to compare between 4% EtOH and normal diet mice within the same genotype category (Wt or ALDH2*2 KI), based on ECGs, western blotting, immuno-confocal, and ex vivo electrophysiological (path clamp or optical mapping) data. Analyses were performed using GraphPad Prism 8 (GraphPad, La Jolla, CA, USA). Statistical analysis was two-tailed and significance was set at *p* < 0.05.

### Reporting summary

Further information on research design is available in the Nature Portfolio Reporting Summary linked to this article.

## Supplementary information

Supplementary Information

Description of Additional Supplementary Files

Supplementary Movie 1

Supplementary Movie 2

Supplementary Movie 3

Supplementary Movie 4

Supplementary Data 1

Reporting Summary

## Data Availability

Requests to access the dataset from qualified researchers trained in human subject confidentiality protocols may be sent to MacKay Memorial Hospital Institutional Data Access/Ethics Committee for researchers (Institutional Review Board Contact information: MacKay Memorial Hospital. Address: No. 92, Sec. 2, Zhongshan N. Rd., Taipei City 10449, Taiwan. TEL: 02-25433535#3486~3488, E-mail: mmhirb82@gmail.com). Source data underlying figures are provided in Supplementary Data 1, and uncropped versions of blots are provided in Supplementary Figs. 11–15. Additional data supporting the findings of this study are available from the corresponding author upon request.
