# Supplementary material for: A Common East Asian aldehyde dehydrogenase 2*2 variant promotes ventricular arrhythmia with chronic light-to-moderate alcohol use in mice
Source: Commun Biol. 2023 Jun 6;6:610. doi: 10.1038/s42003-023-04985-x (PMC10244406; doi:10.1038/s42003-023-04985-x)
Supplement: Supplementary file 1 — Supplementary Information [file 42003_2023_4985_MOESM1_ESM.pdf]

# Supplementary Information

## A Common East Asian Aldehyde Dehydrogenase 2*2 Variant Promotes Ventricular Arrhythmia with Chronic Light-to-Moderate Alcohol Use in Mice


### Authors

An-Sheng Lee[1,2], Yen-Ling Sung[3,4], Szu-Hua Pan[5,6,7], Kuo-Tzu Sung[1,8], Cheng-Huang Su[1,8], Shiao-Li Ding[9], Ying-Jui Lu[9], Chin-Ling Hsieh[9], Yun-Fang Chen[1], Chuan-Chuan Liu[10], Wei-Yu Chen[1], Xuan-Ren Chen[5], Fa-Po Chung[11,12], Shih-Wei Wang[1,13], Che-Hong Chen[14], Daria Mochly-Rosen[14], Chung-Lieh Hung[1,8,13]*, Hung-I Yeh[1,8]*, Shien-Fong Lin[3]

**Affiliations**

1 Departments of Internal Medicine, Mackay Medical College, New Taipei, Taiwan;

2 Division of Cardiovascular Medicine, China Medical University Hospital, Taichung, Taiwan;

3 Institute of Biomedical Engineering, College of Electrical and Computer Engineering, National Yang Ming Chiao Tung University, Hsinchu, Taiwan;

4 Graduate Institute of Biomedical Optomechatronics, Taipei Medical University, Taipei, Taiwan;

5 Graduate Institute of Medical Genomics and Proteomics, College of Medicine, National Taiwan University, Taipei, Taiwan;

6 Genome and Systems Biology Degree Program, National Taiwan University and Academia Sinica, Taipei, Taiwan;

7 Doctoral Degree Program of Translational Medicine, National Taiwan University, 100, Taipei, Taiwan;

8 Division of Cardiology, Departments of Internal Medicine, MacKay Memorial Hospital, Taipei, Taiwan;

9 Department of Medical Research, MacKay Memorial Hospital, New Taipei, Taiwan;

10 Department of Physiology Examination, MacKay Memorial Hospital, New Taipei, Taiwan;

11 Heart Rhythm Center and Division of Cardiology, Department of Medicine, Taipei Veterans General Hospital, Taipei, Taiwan;

12 Department of Medicine, National Yang Ming Chiao Tung University, School of Medicine, Taipei, Taiwan;

13 Institute of Biomedical Sciences, Mackay Medical College, New Taipei, Taiwan;

14 Department of Chemical and Systems Biology, Stanford University, School of Medicine, Stanford, California, U.S.A.

**Reprint requests and correspondence to Chung-Lieh Hung, MD, MSc, Ph.D; Hung-I Yeh, MD, Ph.D**

Division of Cardiology, Department of Internal Medicine, Mackay Memorial Hospital, Mackay Medical College, New Taipei City, Taiwan

Tel: +886-2-25433535; Fax: +886-2-25433535 ext: 2459;

E-mail: jotaro3791@gmail.com; hiyeh@ms1.mmh.org.tw

# Supplemental Figures

**Supplementary Figure 1**

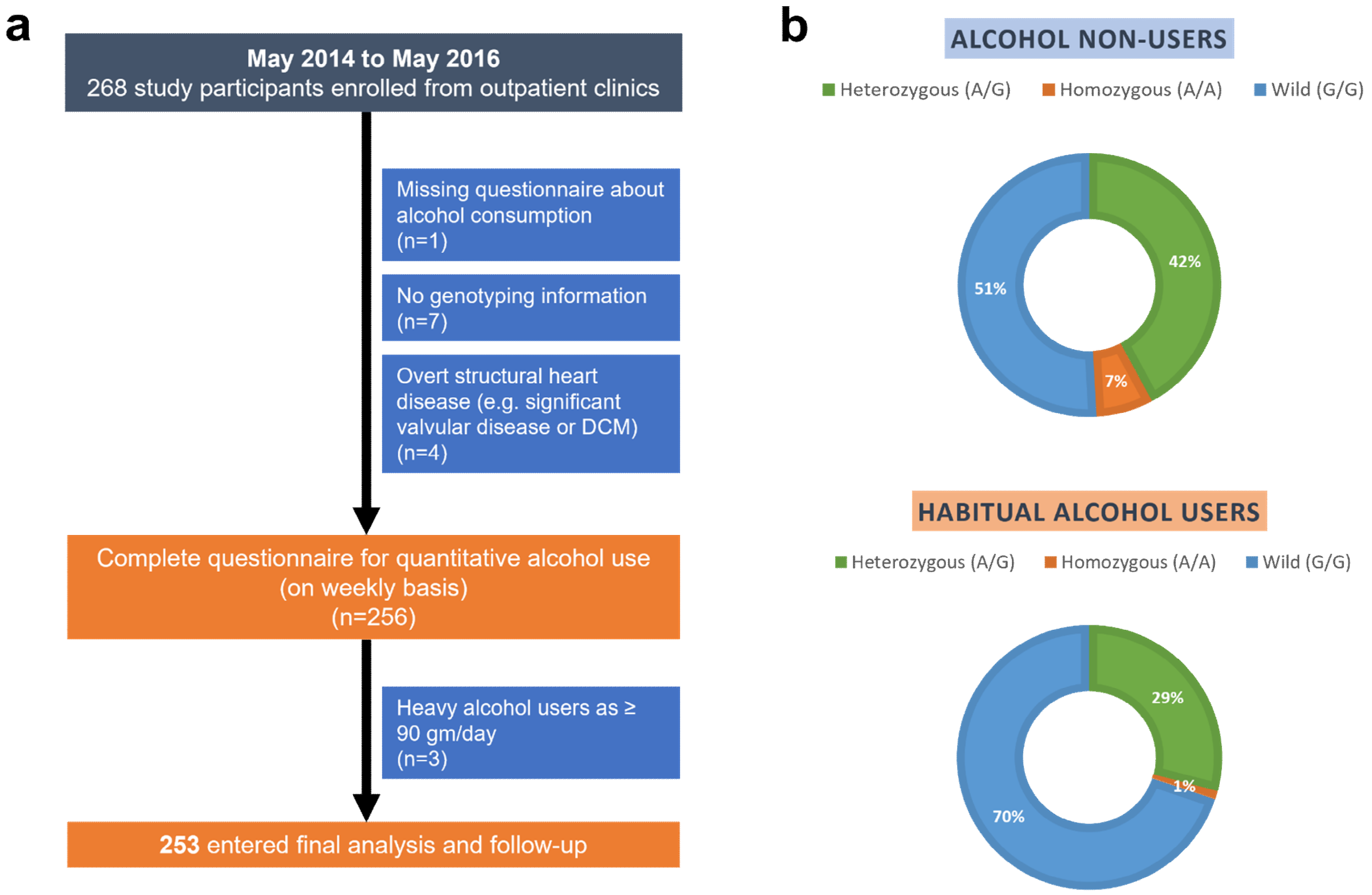

**Supplementary Figure 1. Flowchart for study enrollment, exclusion criteria and *ALDH2* rs671 genotyping results and distribution in our human study.**

* $p < 0.05$ vs. *ALDH2* wild type (G/G, rs671) group; $^{\#}$ $p < 0.05$ vs. *ALDH2* variant (G/A, rs671 or A/A, rs671) group.

**Supplementary Figure 2**

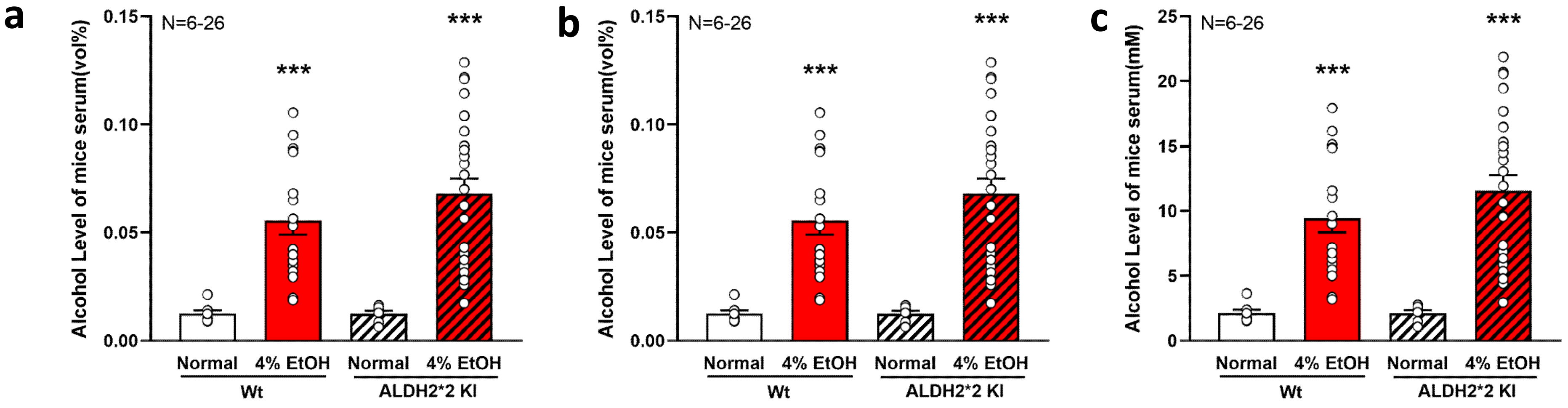


**Supplementary Figure 2. Effect of *ALDH2*2* knock-in (KI) on blood alcohol level (BAC) 1 hour post normal diet or 4% EtOH diet (a-c) and relationships of BAC with QTc in 4% EtOH-treated mice (d).**

Data are presented as mg/dl (a), vol% (b) and mmol (mM) (c). Substantially higher BAC levels were observed in both EtOH-fed mice compared to those of respective normal diet control groups (a-c). More prominent of QTc prolongation was observed with higher BAC levels in EtOH-fed *ALDH2*2* KI mice (r = 0.72, p < 0.001) compared to EtOH-fed Wt mice (r = 0.58, p = 0.01) ($p_{interaction}$: 0.035). Data are expressed as the mean±SE for normally distributed variables. **p* < 0.05, ** *p* < 0.01, *** *p* < 0.001 for any 4% EtOH vs normal diet control within each genotype group (*ALDH2*2* KI or Wt).

Supplementary Figure 3

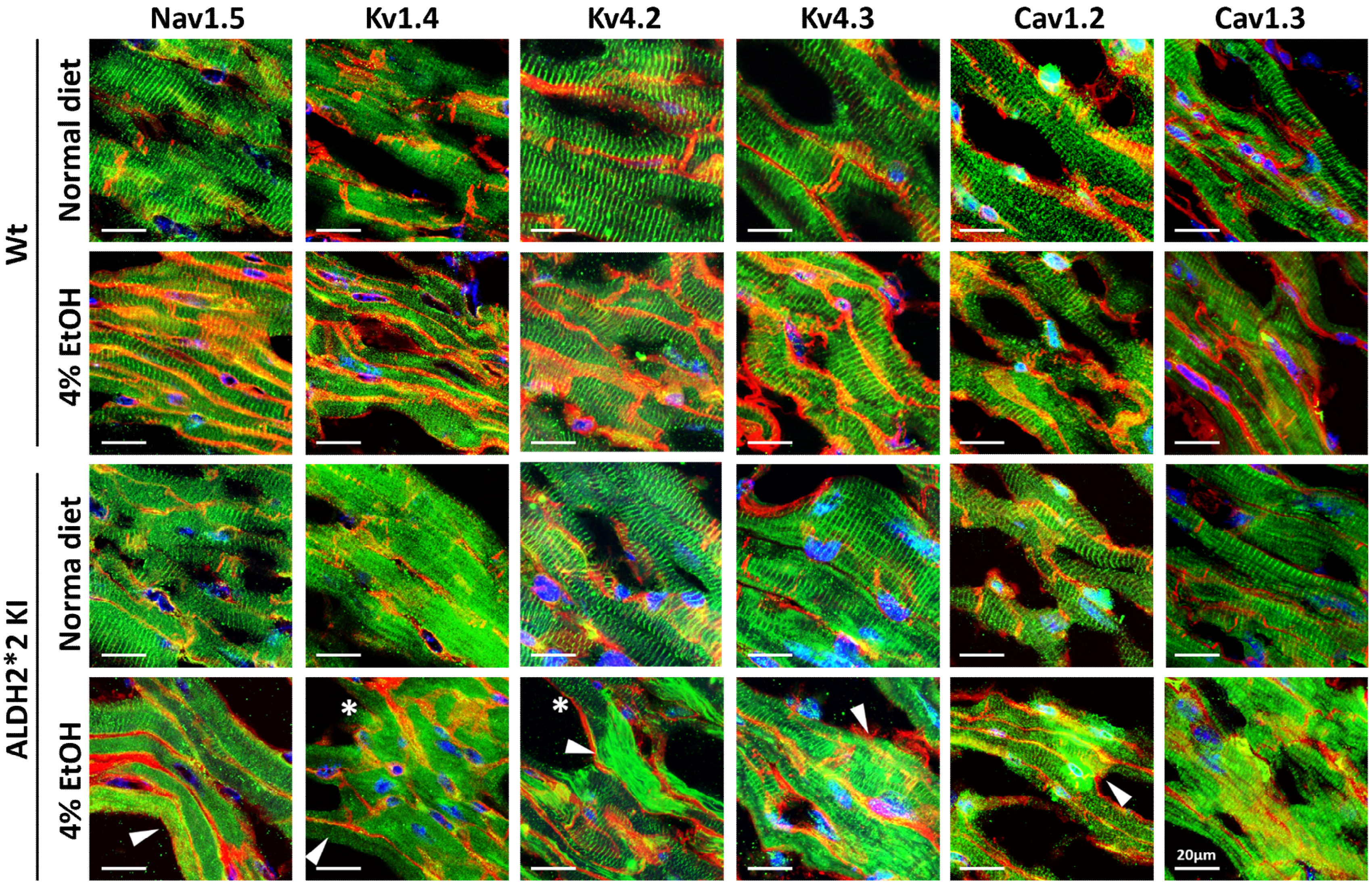

**Supplementary Figure 3**. **Ion channel protein expression patterns among different mouse groups are illustrated.**

Ventricular cardiomyocytes were stained for Nav1.5 (green), Cav1.2 (green), Cav1.3 (green), Kv1.4 (green), Kv4.2 (green), Kv4.3 (green), and WGA (red), along with DAPI staining (blue). Disorganized staining of Nav1.5 (white arrowhead), attenuated staining and loss of striation of Kv1.4 (white arrowhead and asterisk labeled), irregular aggregations and attenuated staining of Kv4.2 (white arrowhead and asterisk labeled), accentuated staining of Kv4.3 (white arrowhead) and both accentuated Cav1.2 and Cav1.3 though altered aggregation in Cav1.2 (white arrowhead) were more prominent in EtOH-treated *ALDH2*2* KI mice. Scale bar: 20 μm.

Supplementary Figure 4

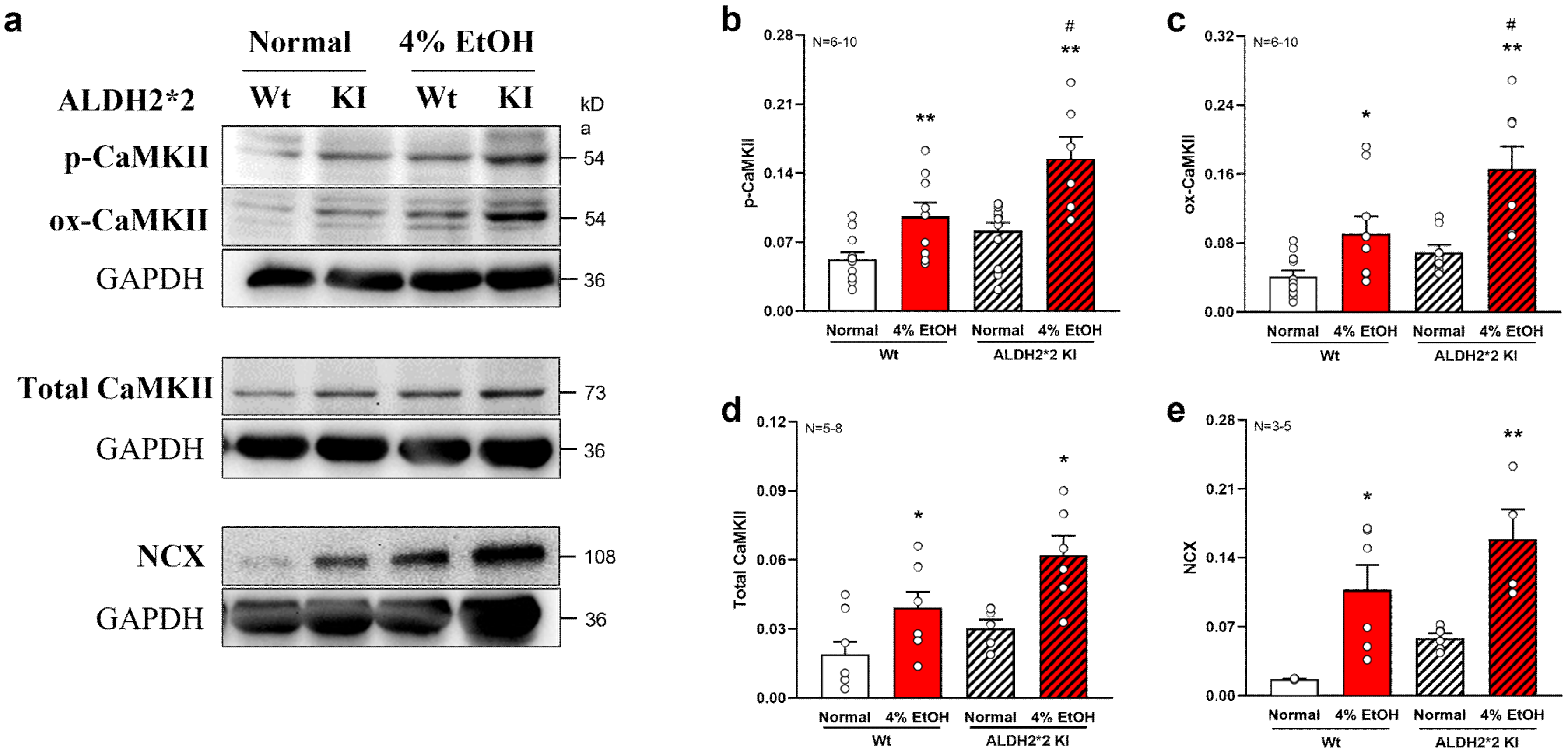

**Supplementary Figure 4**. **Densitometric analysis of heart tissue CaMKII (total and oxidized [ox-CaMKII] forms) and NCX are illustrated.**

Western blotting (a) showing both CaMKII (total and phosphorylated/oxidized forms) (b-d) and NCX (e) were upregulated in EtOH-treated KI mice compared to those in their respective normal diet control groups irrespective of genotypes (Wt and *ALDH2*2* KI), with CaMKII (both phosphorylated/oxidized forms) being the most prominently upregulated proteins in EtOH-treated *ALDH2*2* KI mice. Data are expressed as the mean±SE for normally distributed variables. * $p < 0.05$, ** $p < 0.01$, *** $p < 0.001$ for any 4% EtOH vs normal diet control within each genotype group (*ALDH2*2* KI or Wt); $^{\#} p < 0.05$, $^{\#\#} p < 0.01$, $^{\#\#\#} p < 0.001$ for 4% EtOH *ALDH2**2 KI vs 4% EtOH Wt.

**Supplementary Figure 5**

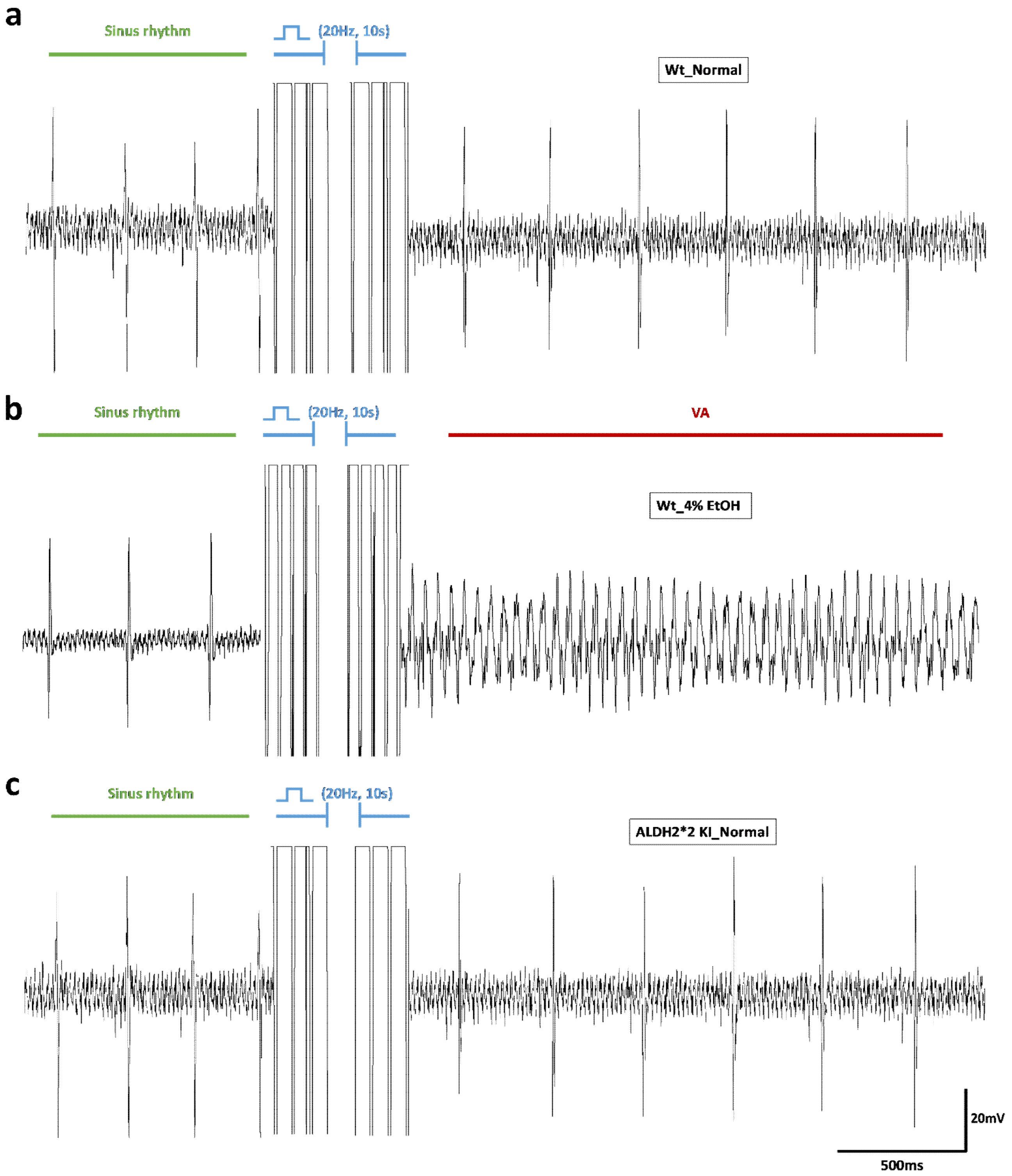


**Supplementary Figure 5. Vulnerability of ventricular arrhythmia (VA) after programmed electrical stimulation (PES) induction.**

Example of surface ECG recording. VA, ventricular arrhythmia; PES, programmed electrical stimulation.

Supplementary Figure 6

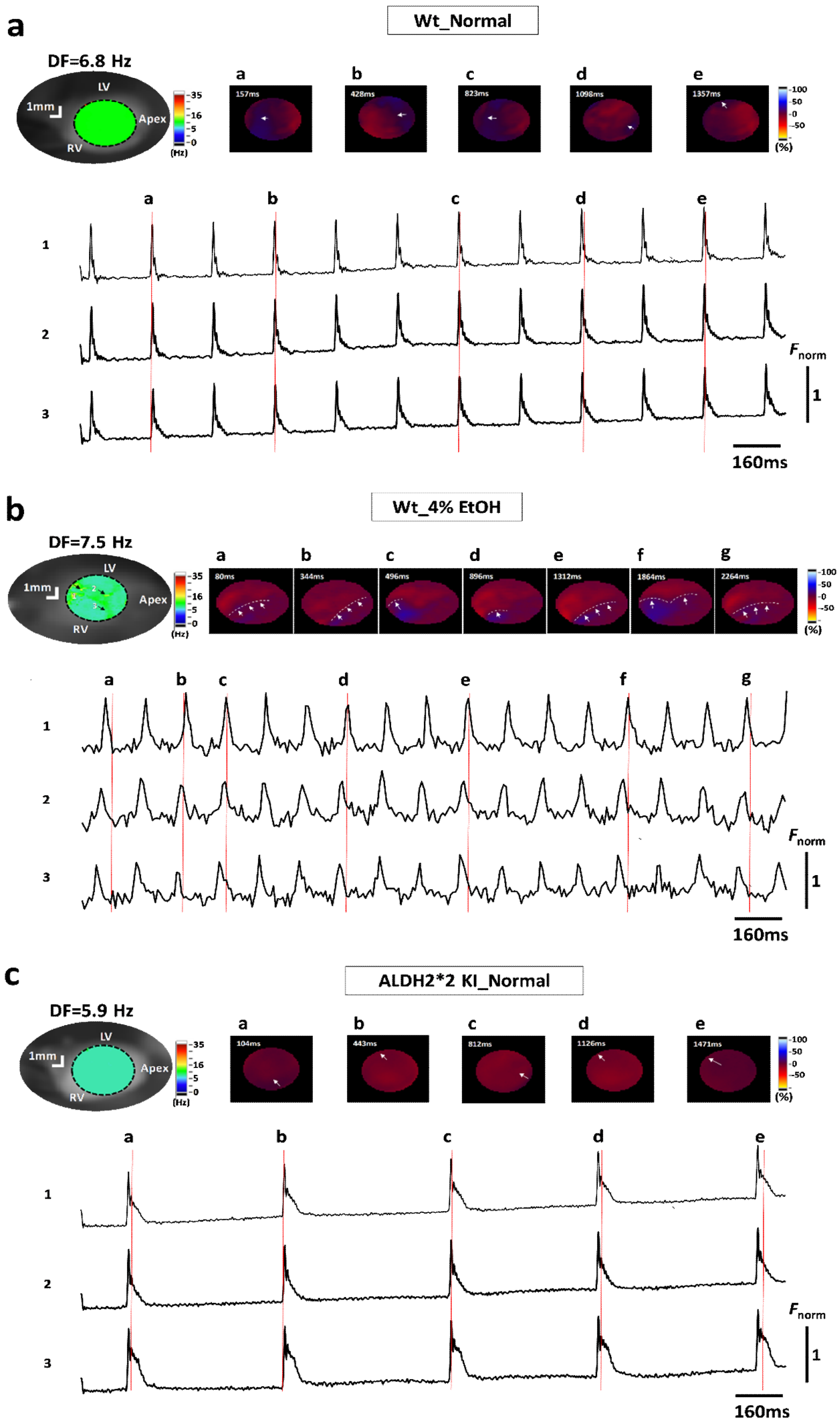

**Supplementary Figure 6. Optical mapping and electrical signal propagation.**

Dominant frequency (DF) points and snapshots of spiral waves corresponding to the time domain at a given point. DF, dominant frequency.

**Supplementary Figure 7**

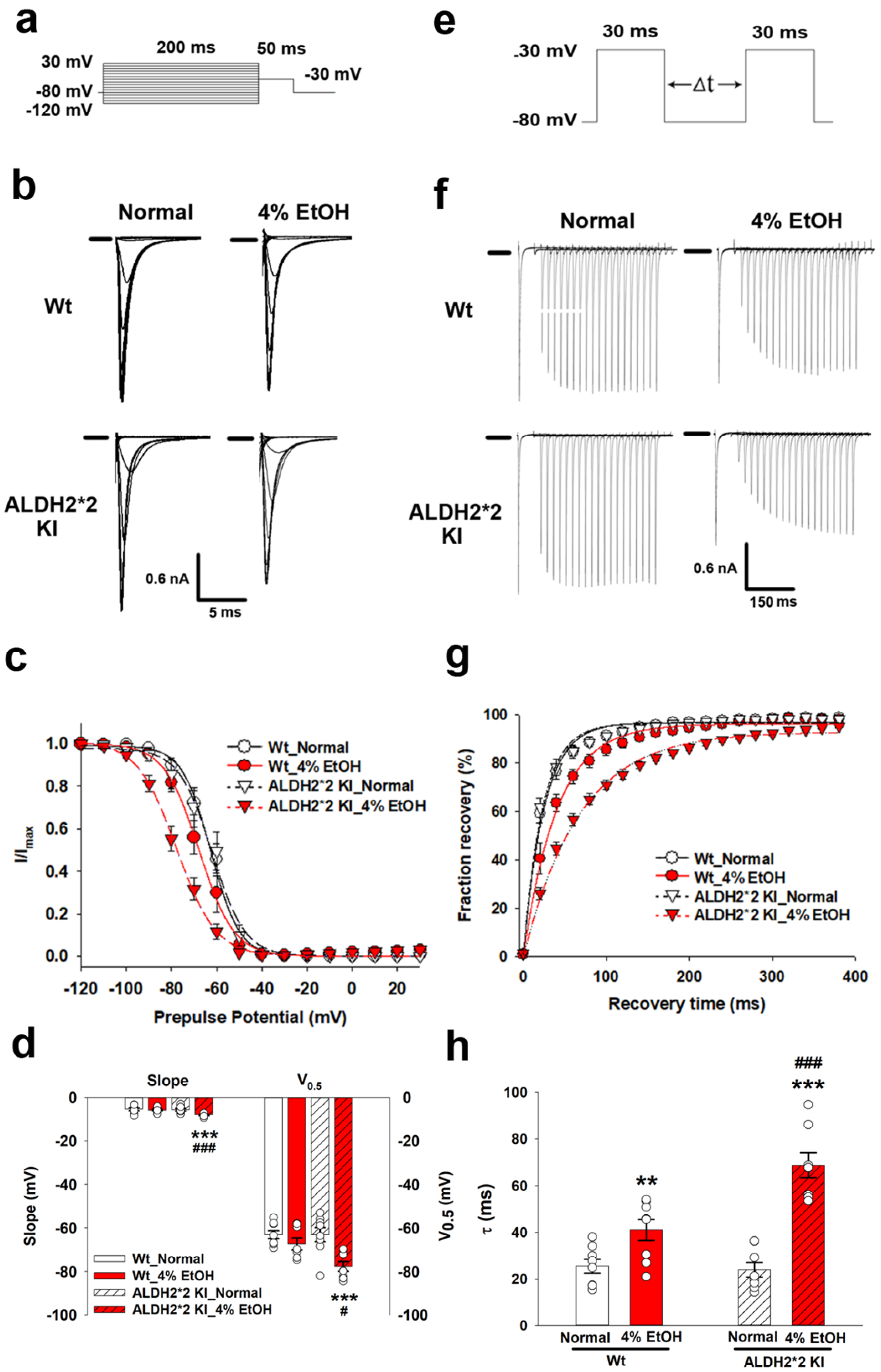

**Supplementary Figure 7. Comparisons of the gating properties of $I_{Na}$ inactivation curves and recovery of cardiomyocyte from inactivation in cardiomyocytes of all mice groups.** Different voltage levels of conditioning pre-pulses (-120 to 30 mV) were applied for 200 ms to induce channel inactivation, in order to examine the inactivation of voltage-dependent $I_{Na}$. A second pulse (-30 mV) was then used to depolarize the membrane potential for 50 ms (a). The amplitude of $I_{Na}$ was then normalized to the maximal current amplitude (b), and the traces were fitted using the Boltzmann equation model (c). The results showed that the $I_{Na}$ inactivation curve in the cardiomyocytes of *ALDH2*2* KI mice treated with 4% EtOH was substantially left-shifted with a steeper slope and $V_{0.5}$ observed compared to normal diet *ALDH2*2* KI mice or EtOH-treated Wt mice (both $p < 0.01$, respectively) (d), indicating diversely affected $I_{Na}$ kinetics. However, EtOH-treated Wt mice only showed a slight yet non-significant shift in the inactivation curve of cardiomyocytes compared to normal diet Wt mice (d). On the other hand, recovery of $I_{Na}$ from inactivation was determined using a typical paired-pulse protocol. After a -80 mV holding potential, 2 identical -30 mV pulses for 30 ms were separated in each intervals (0-380 ms) (e). Currents against intervals between paired-pulses were generated and plotted (f) and the traces were fitted by a single exponential equation (g). The recovery curves from inactivation in cardiomyocytes of both EtOH-treated (Wt and *ALDH2*2* KI) mice were significantly right-shifted with increased time constant ($\tau$) when compared to those of their respective normal diet control groups ($p = 0.01$, $p < 0.01$, respectively; h), indicating diversely altered AP kinetics. Likewise, representative $\tau$ was significantly higher in the cardiomyocytes of 4% EtOH-treated (Wt and *ALDH2*2* KI) mice than those of their respective normal diet control groups (both $p < 0.01$), with EtOH-treated *ALDH2*2* KI further showing significantly higher $\tau$ than that of EtOH-treated Wt mice (h). These findings indicated that significantly downregulated $I_{Na}$ was followed by a slower depolarization of the action potential in single cardiomyocyte AP kinetics. n = 8 independent cells in each group. Data are expressed as the mean±SE for normally distributed variables.

**Supplementary Figure 8**

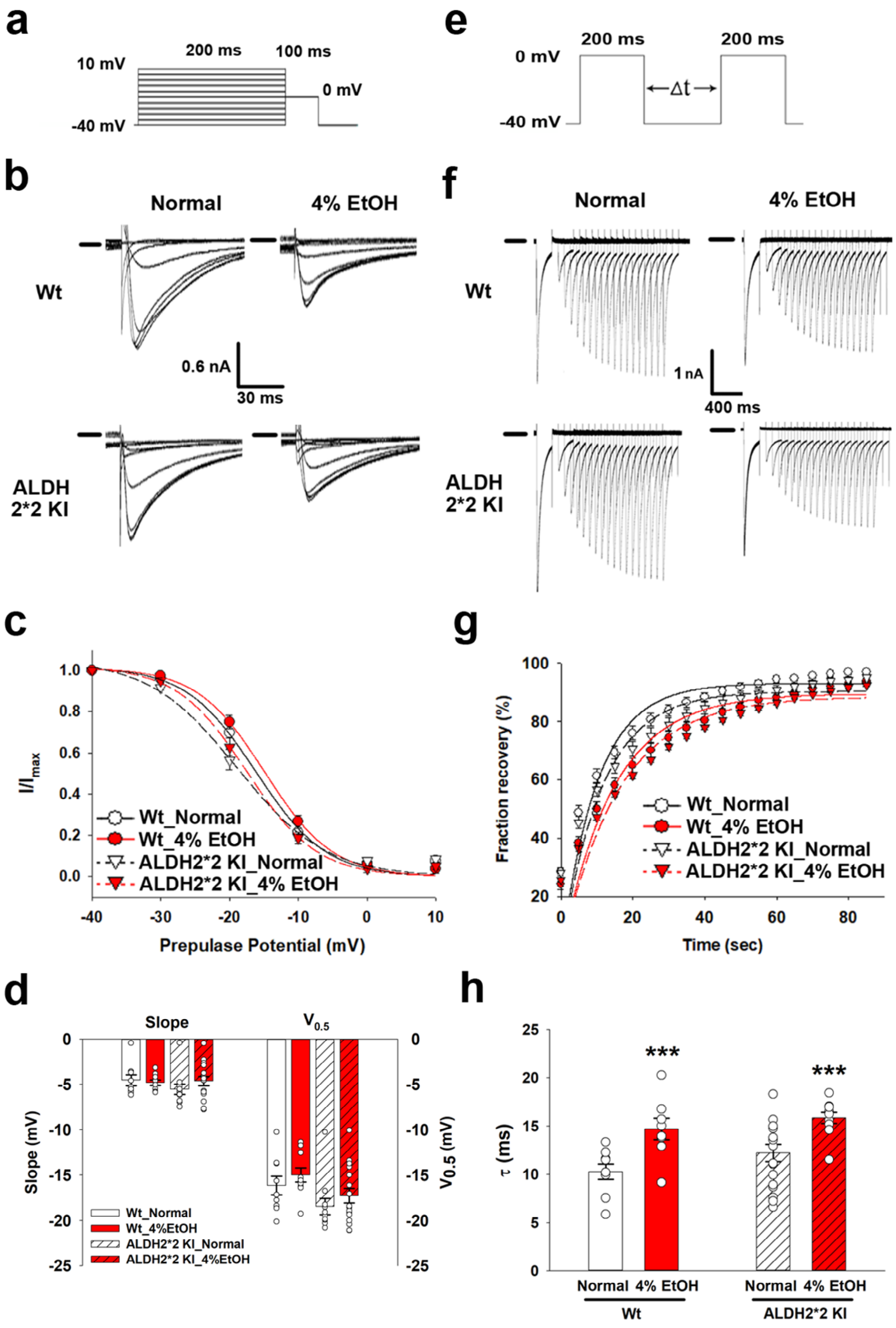

**Supplementary Figure 8. Comparisons of the gating properties of $I_{Ca}$ inactivation curves and recovery of cardiomyocyte from inactivation in cardiomyocytes of all mice groups.** To examine the inactivation of voltage-dependent $I_{Ca}$, different voltage levels of conditioning pre-pulses (-40 to 10 mV) were applied for 200 ms to induce channel inactivation. A second pulse (0 mV) was then applied for 100 ms to depolarize the membrane potential (a). The amplitude of $I_{Ca}$ was then normalized to the maximal current amplitude (b), and the traces were fitted using the Boltzmann equation model (c). No significant changes were observed between the $I_{Ca}$ inactivation curves of the cardiomyocytes of all mice groups (d). Recovery of $I_{Ca}$ from inactivation was determined using a typical paired-pulse protocol. After holding a potential of -40 mV, two identical 200 ms long 0 mV pulses were applied with intervals of 0-85 ms in between (e). Currents against intervals between paired-pulses were plotted (f), and the traces were fitted via a single exponential equation (g). Recovery curves of the cardiomyocytes of both EtOH-treated (Wt and *ALDH2*2* KI) mouse groups were significantly right-shifted with increased time constant ($\tau$) when compared to those of their respective controls ($\tau$; both $p < 0.01$, respectively) (h). n = 9 and 8 independent cells in each group for inactivation and recovery, respectively. Data are expressed as the mean±SE for normally distributed variables.

**Supplementary Figure 9**

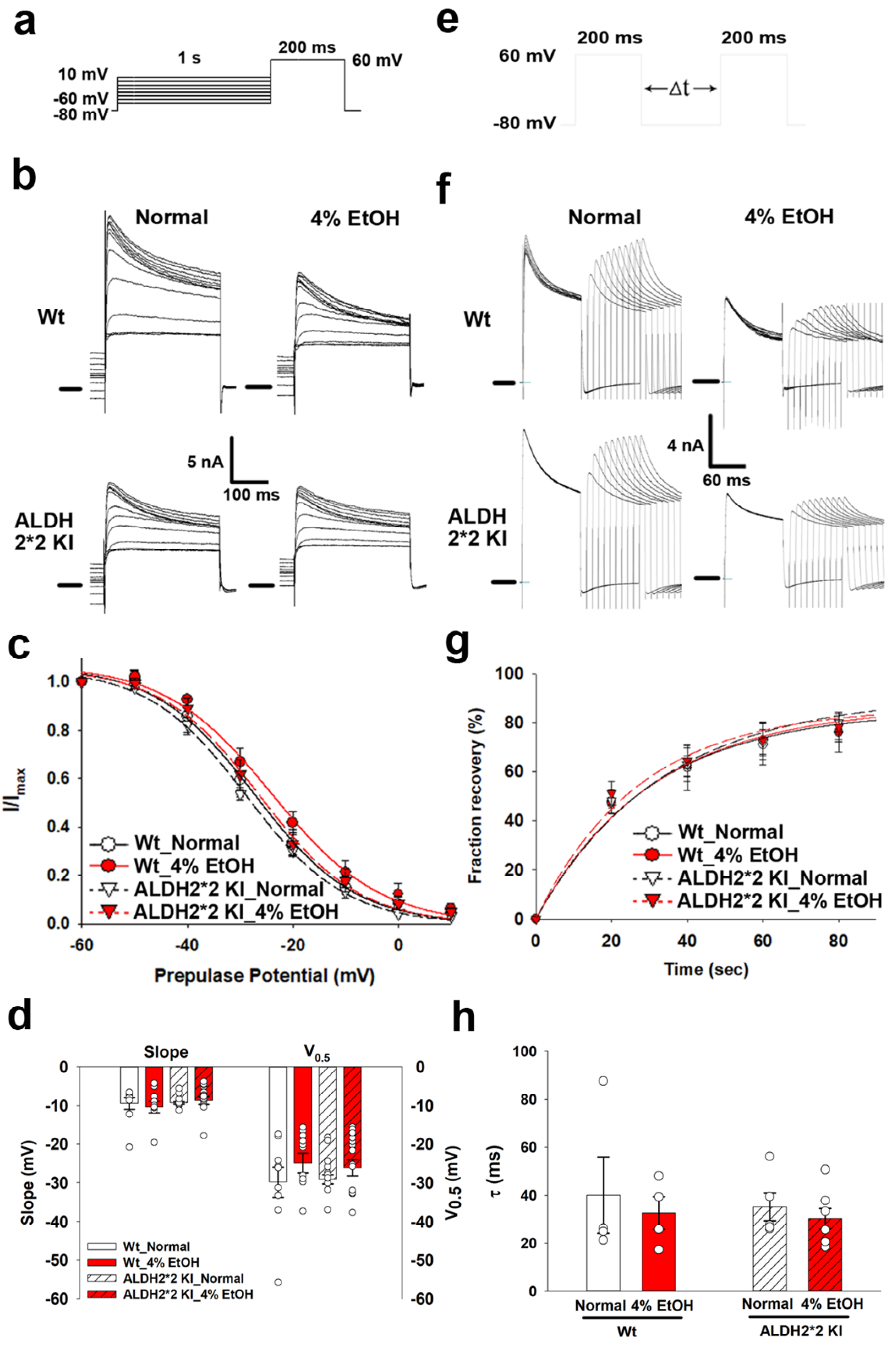

**Supplementary Figure 9. Comparisons of the gating properties of $I_{to}$ inactivation curves and recovery of cardiomyocyte from inactivation in cardiomyocytes of all mice groups.** There were no significant differences between the inactivation curves and recovery of $I_{to}$ from inactivation of the cardiomyocytes in the different mouse groups. n = 9 and 4 independent cells in each group for inactivation and recovery, respectively. Data are expressed as the mean±SE for normally distributed variables.

**Supplementary Figure 10**

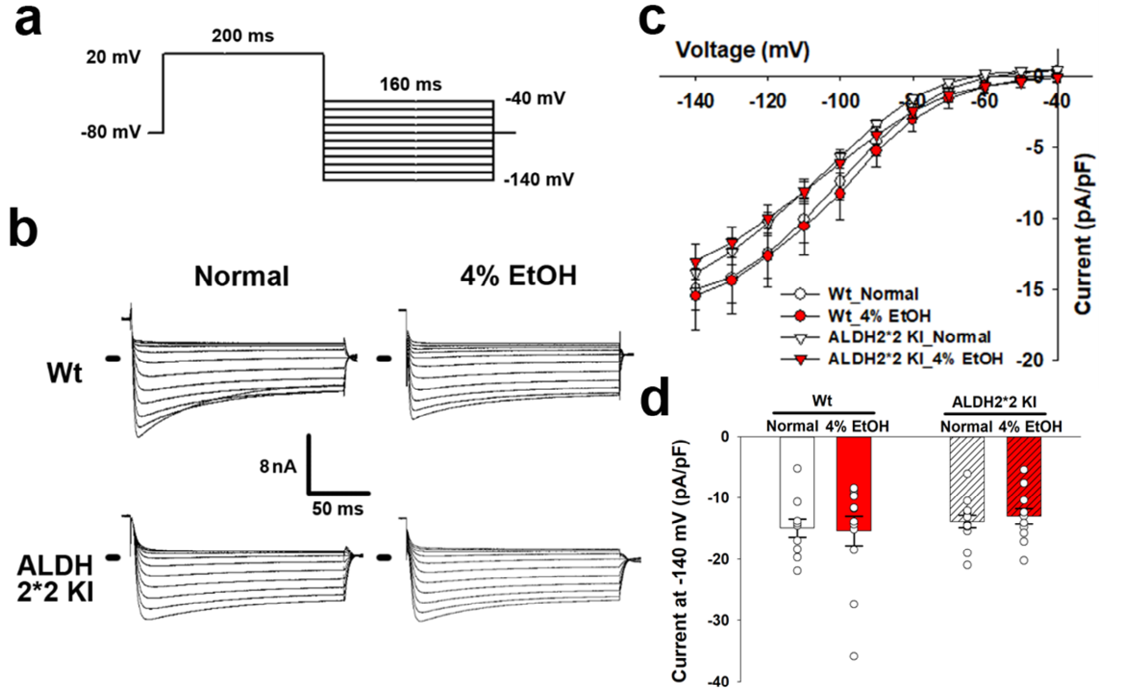


**Supplementary Figure 10. Comparisons of the inward rectifier potassium current (*I*K1) were found to vary among different mice groups (detailed in Supplemental Materials).** $I_{K1}$ is mainly responsible for the stability of the resting membrane potential. It also showed that the $I_{K1}$ in the cardiomyocytes of both EtOH-treated (Wt or *ALDH2*2* KI) mice groups were not significantly different from those of their respective normal diet controls. n = 10 independent cells in each group. Data are expressed as the mean±SE for normally distributed variables.

**Supplementary Figure 11**

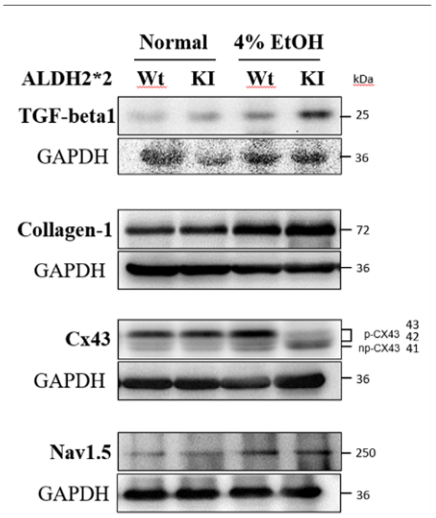


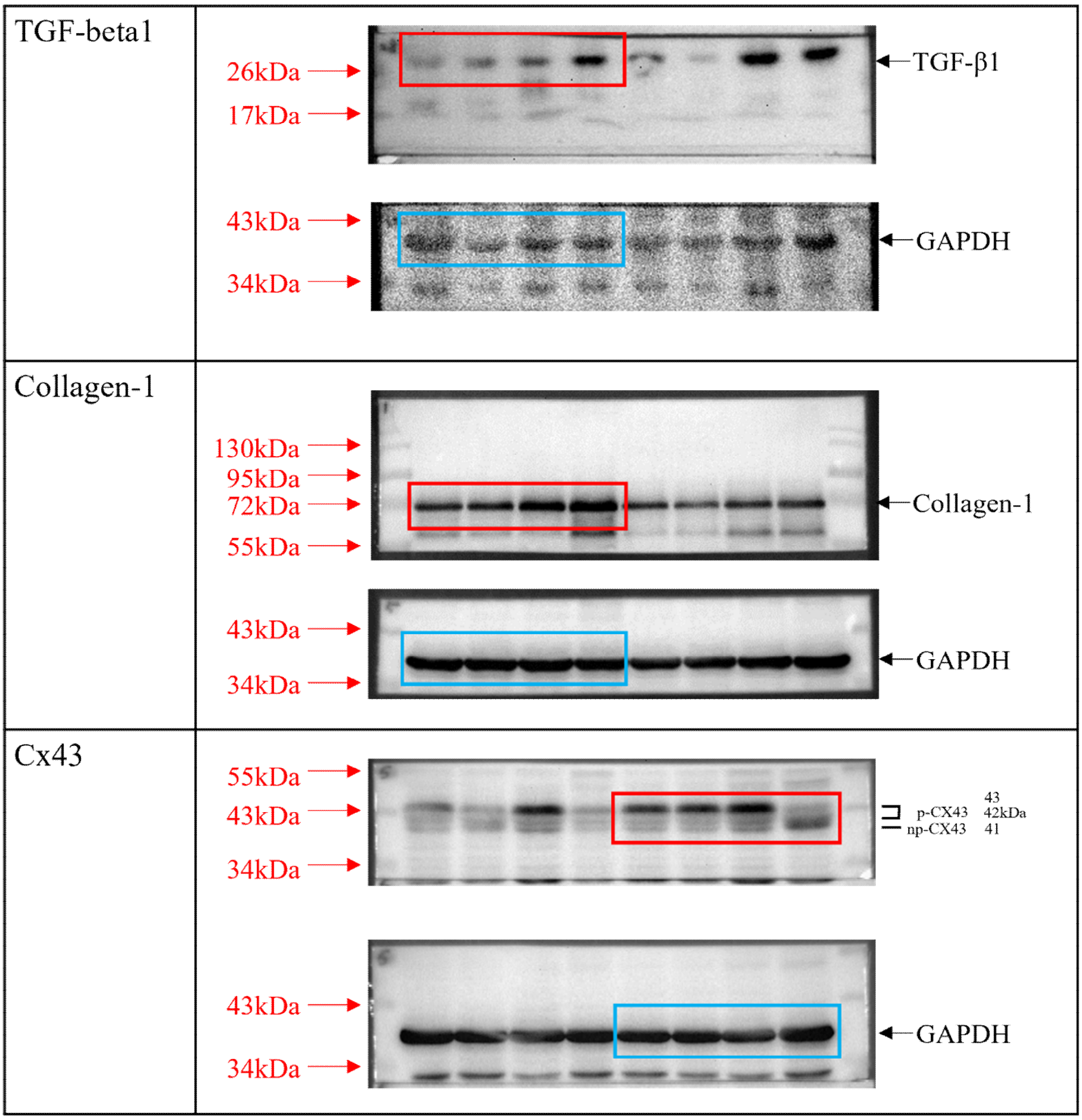

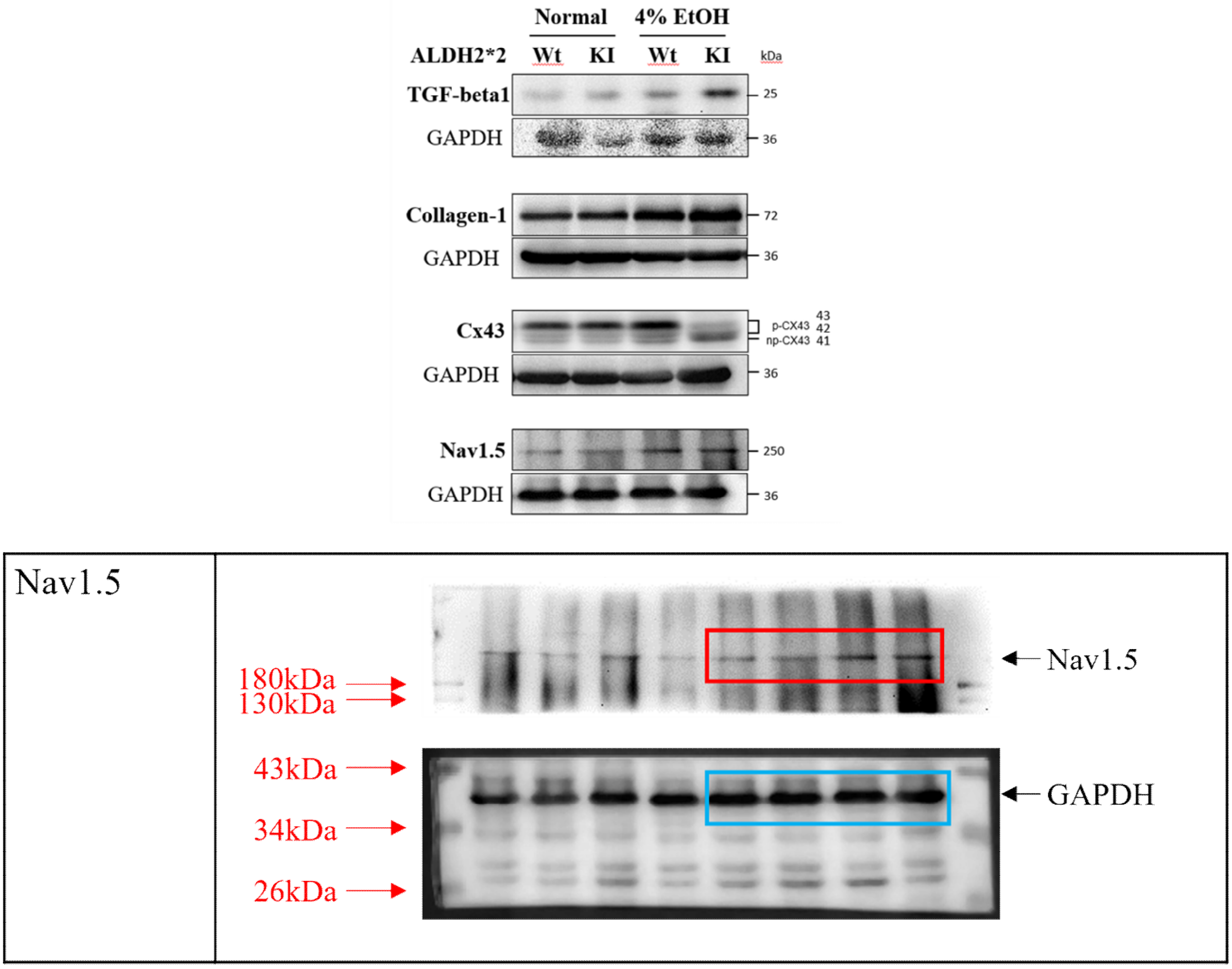


**Supplementary Figure 11. Uncropped original densitometric western blot analysis of heart tissue for TGF-β1/collagen-1, Cx43 (total form), and Nav1.5.**

## Supplementary Figure 12

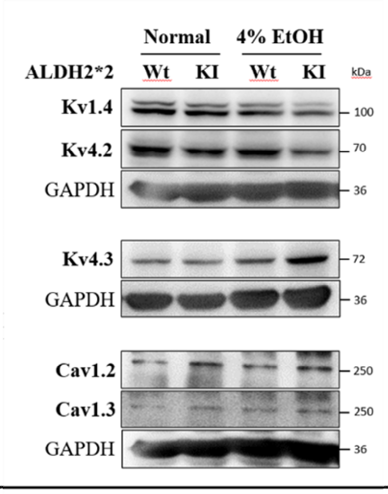


| | |
|---|---|
| Kv1.4 | 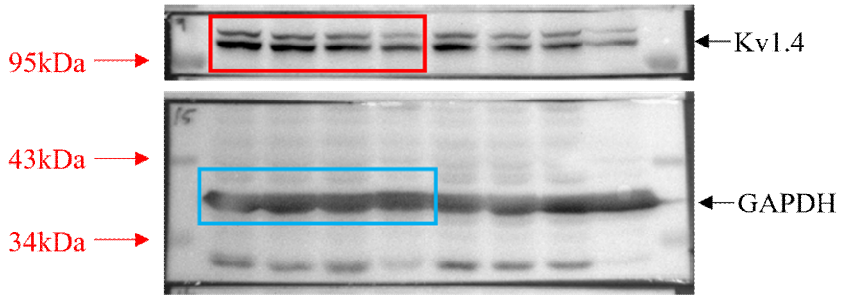 |
| Kv4.2 | 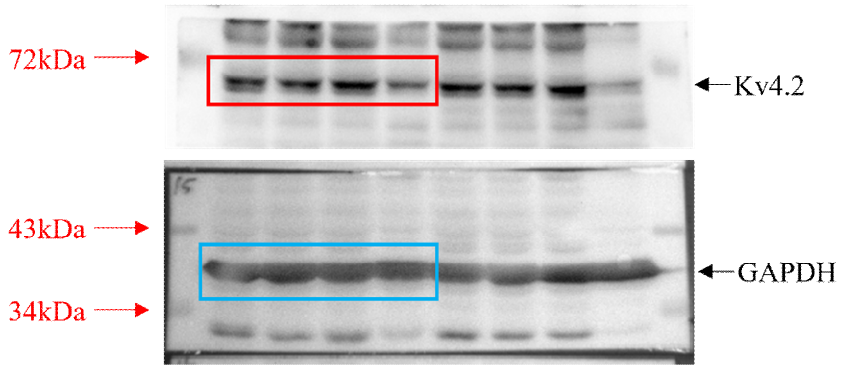 |
| Kv4.3 | 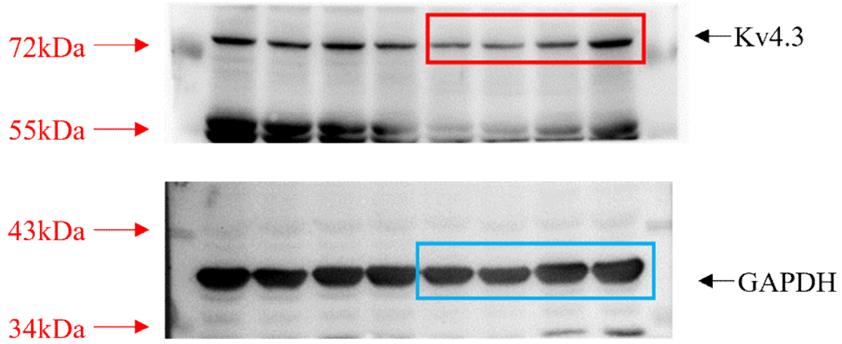 |

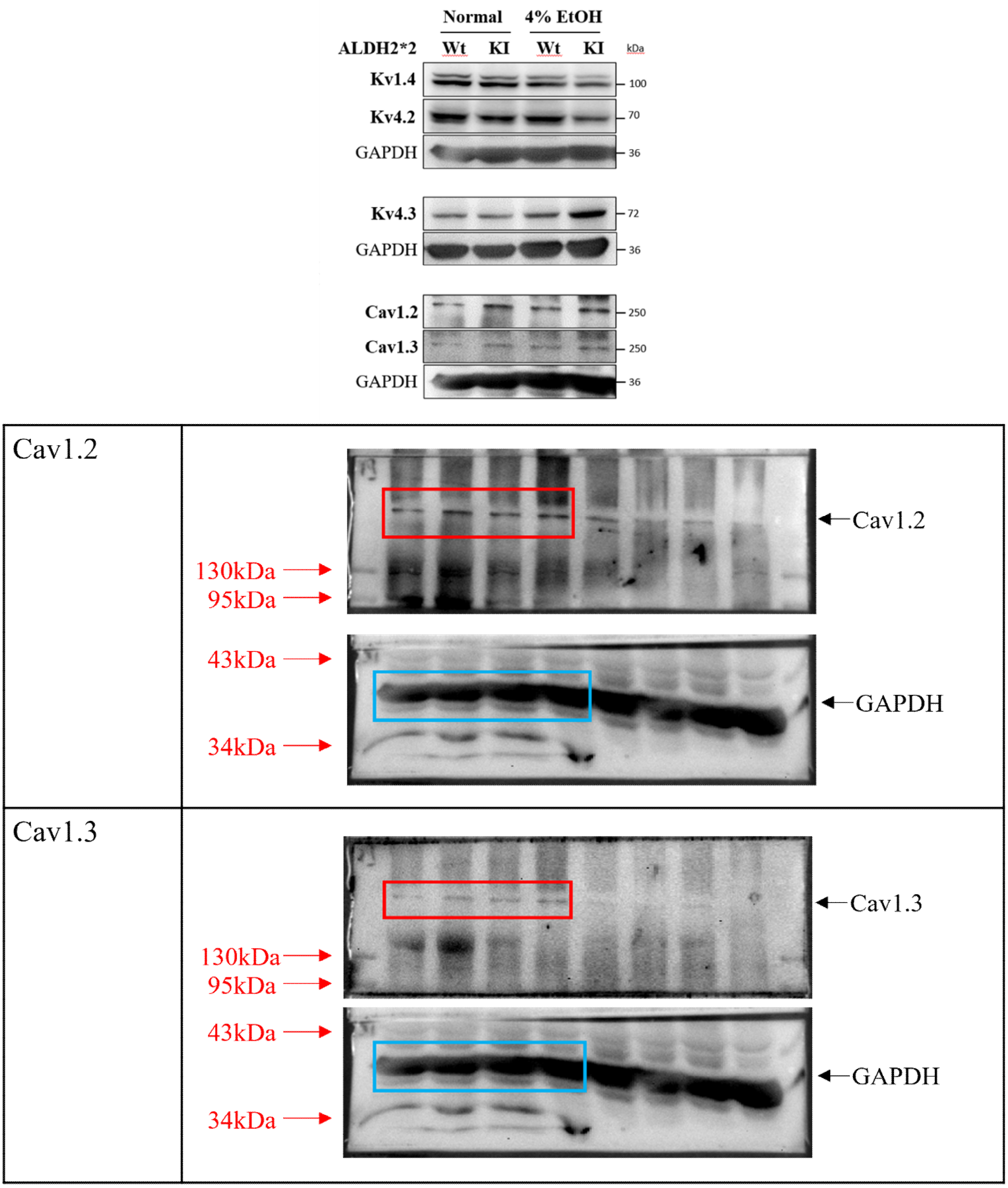


**Supplementary Figure 12. Uncropped original densitometric western blot analysis of heart tissue for Kv1.4, Kv4.2, Kv4.3, Cav1.2, and Cav1.3.**

**Supplementary Figure 13**

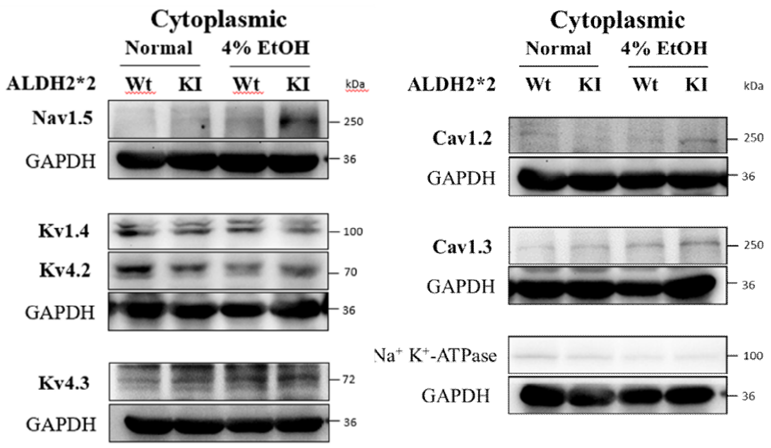


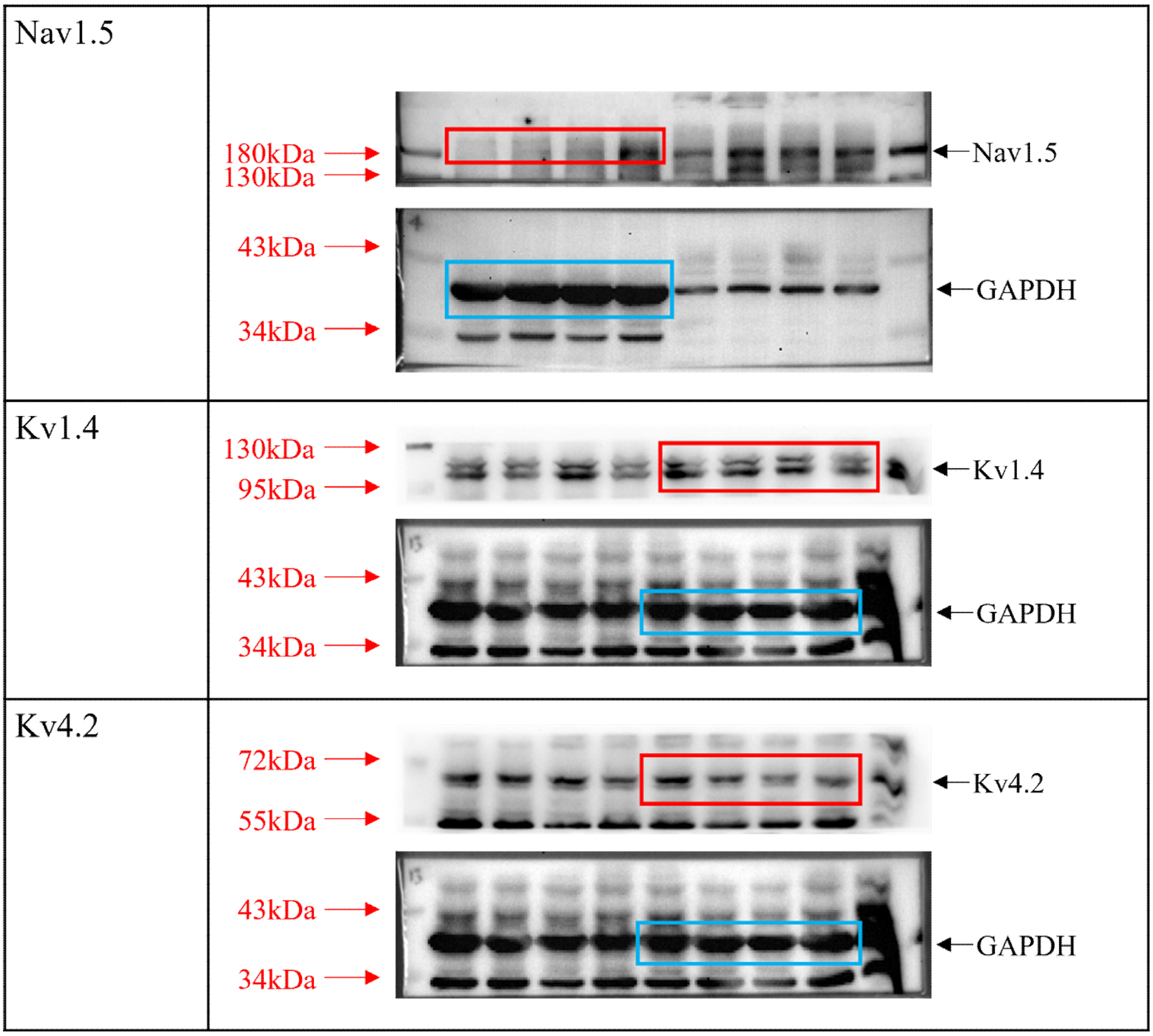

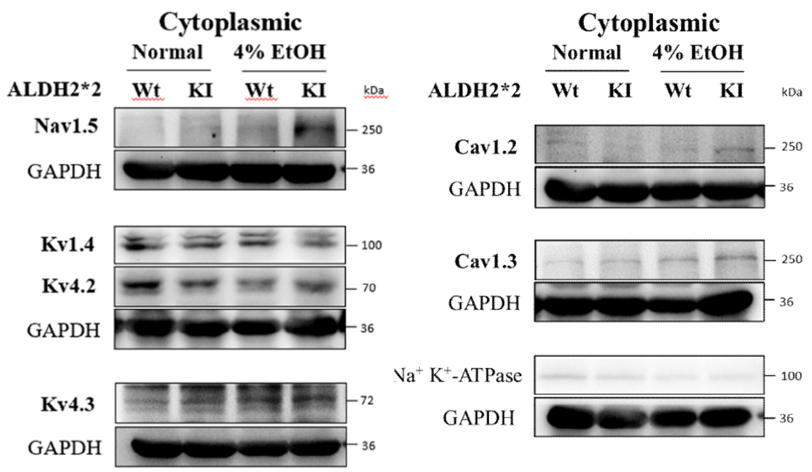


| | |
|---|---|
| Kv4.3 | 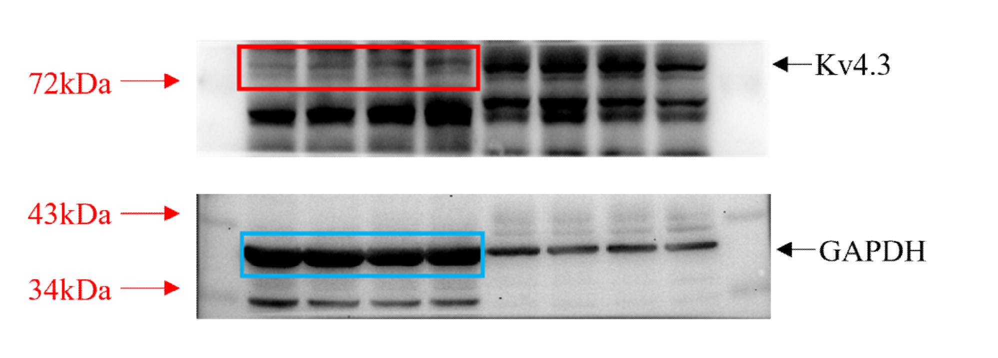  |
| Cav1.2 | 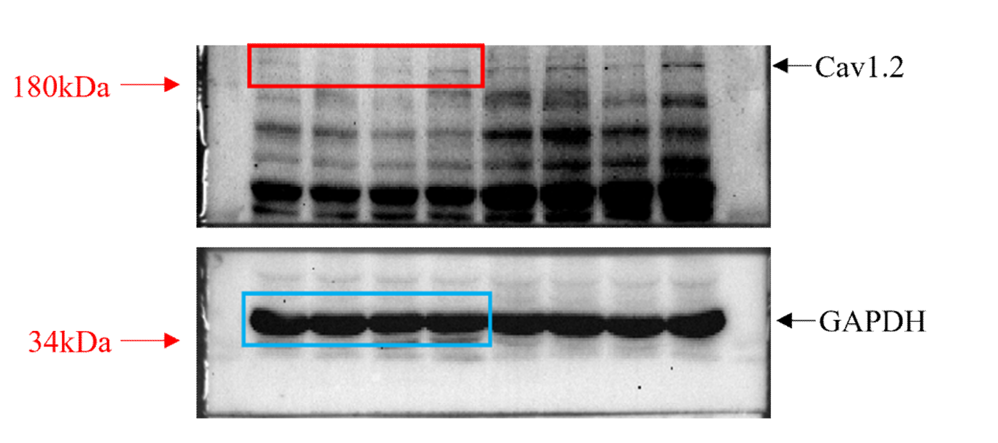  |
| Cav1.3 | 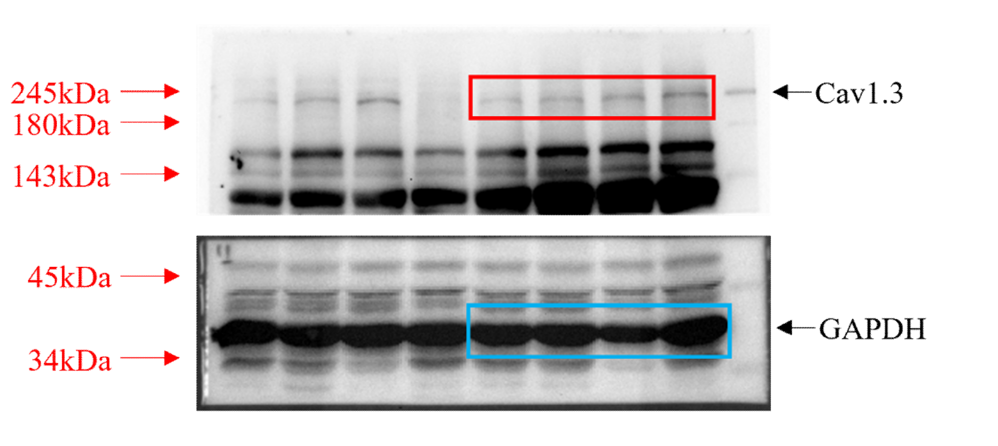  |

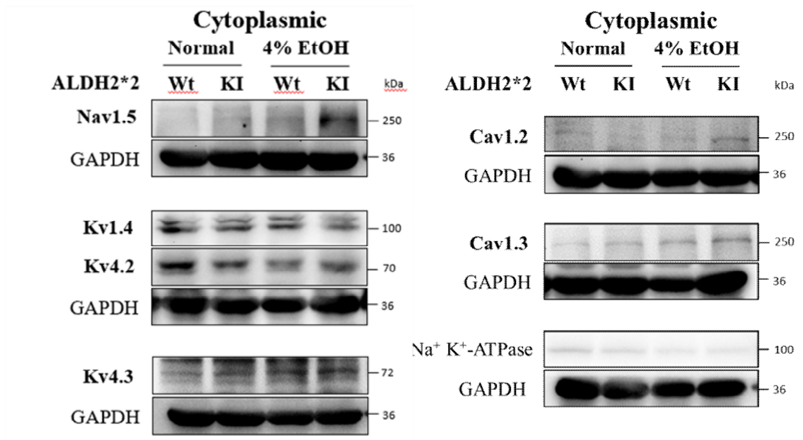


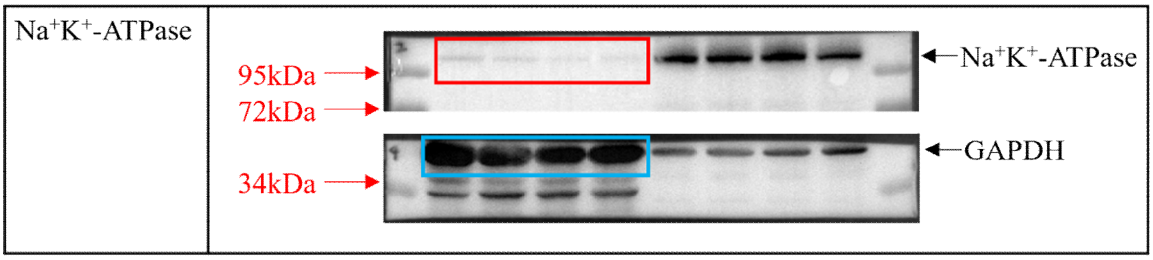


**Supplementary Figure 13. Uncropped original densitometric western blot analysis of heart tissue for cytoplasmic fractions of ion channels expression.**

**Supplementary Figure 13. Uncropped original densitometric western blot analysis of heart tissue for cytoplasmic fractions of ion channels expression.**

**Supplementary Figure 14**

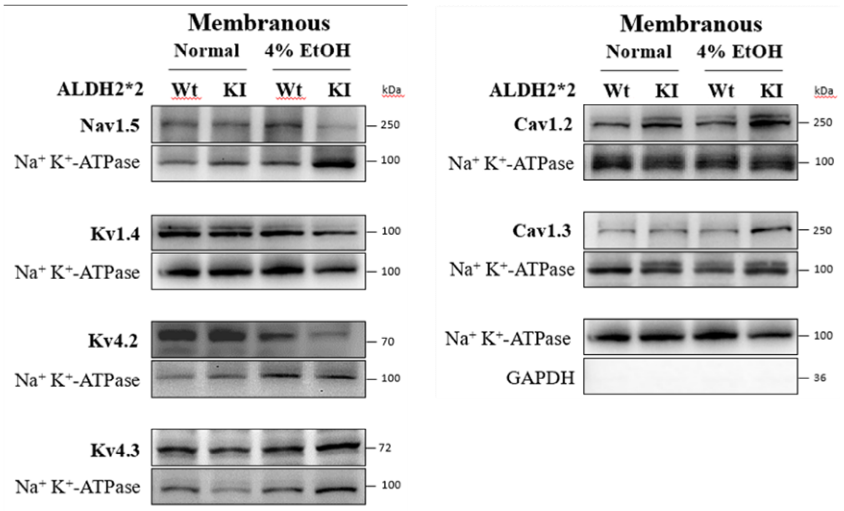


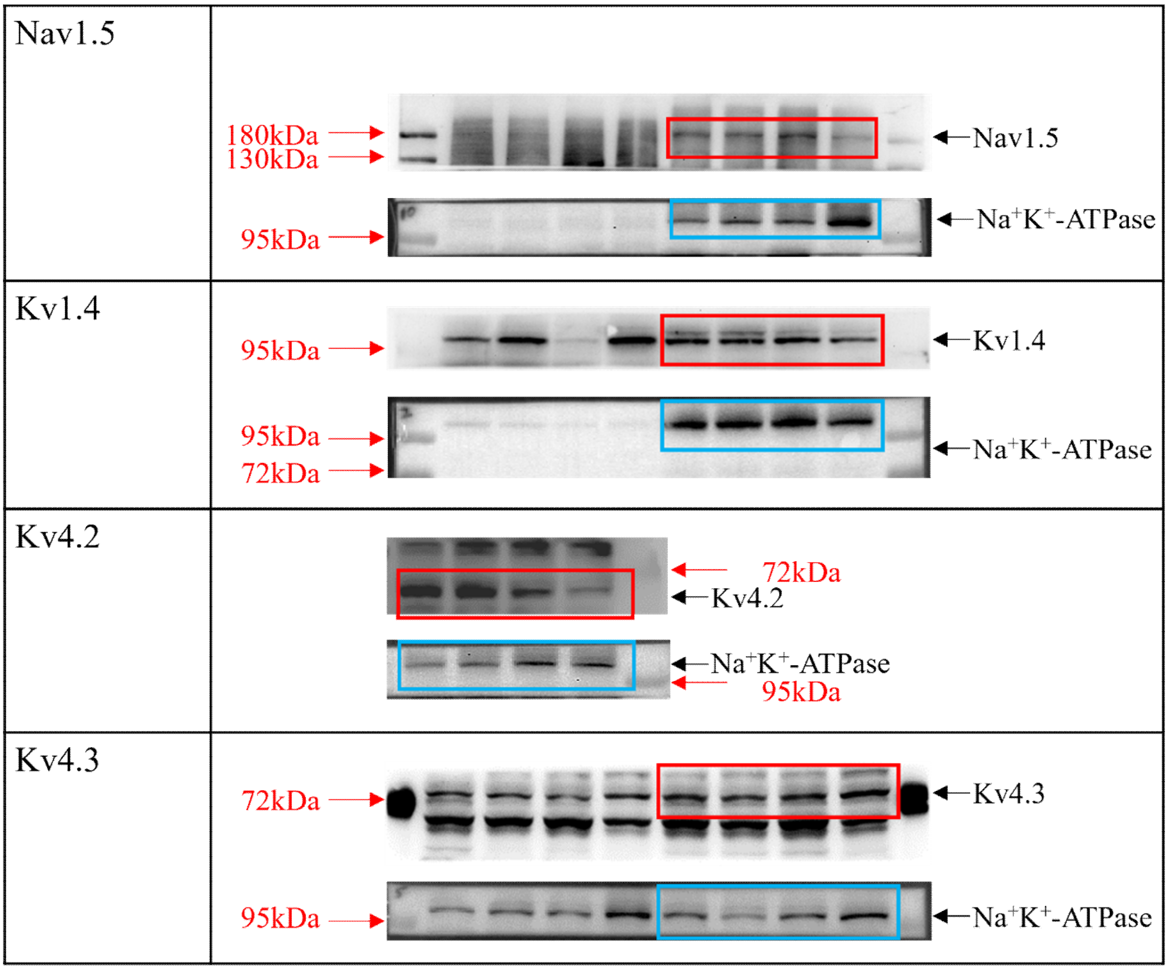

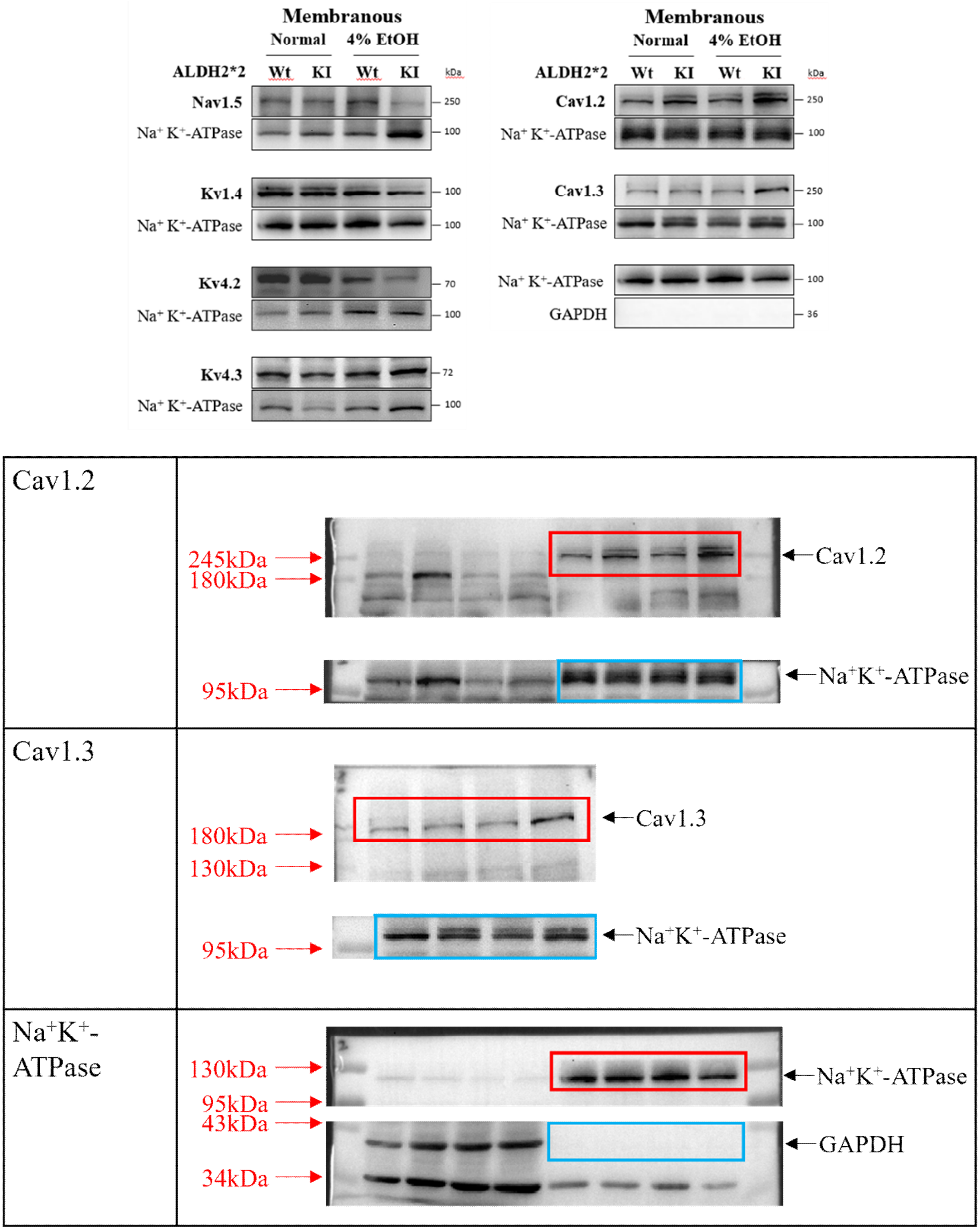


**Supplementary Figure 14. Uncropped original densitometric western blot analysis of heart tissue for membranous fractions of ion channels expression.**

**Supplementary Figure 15**

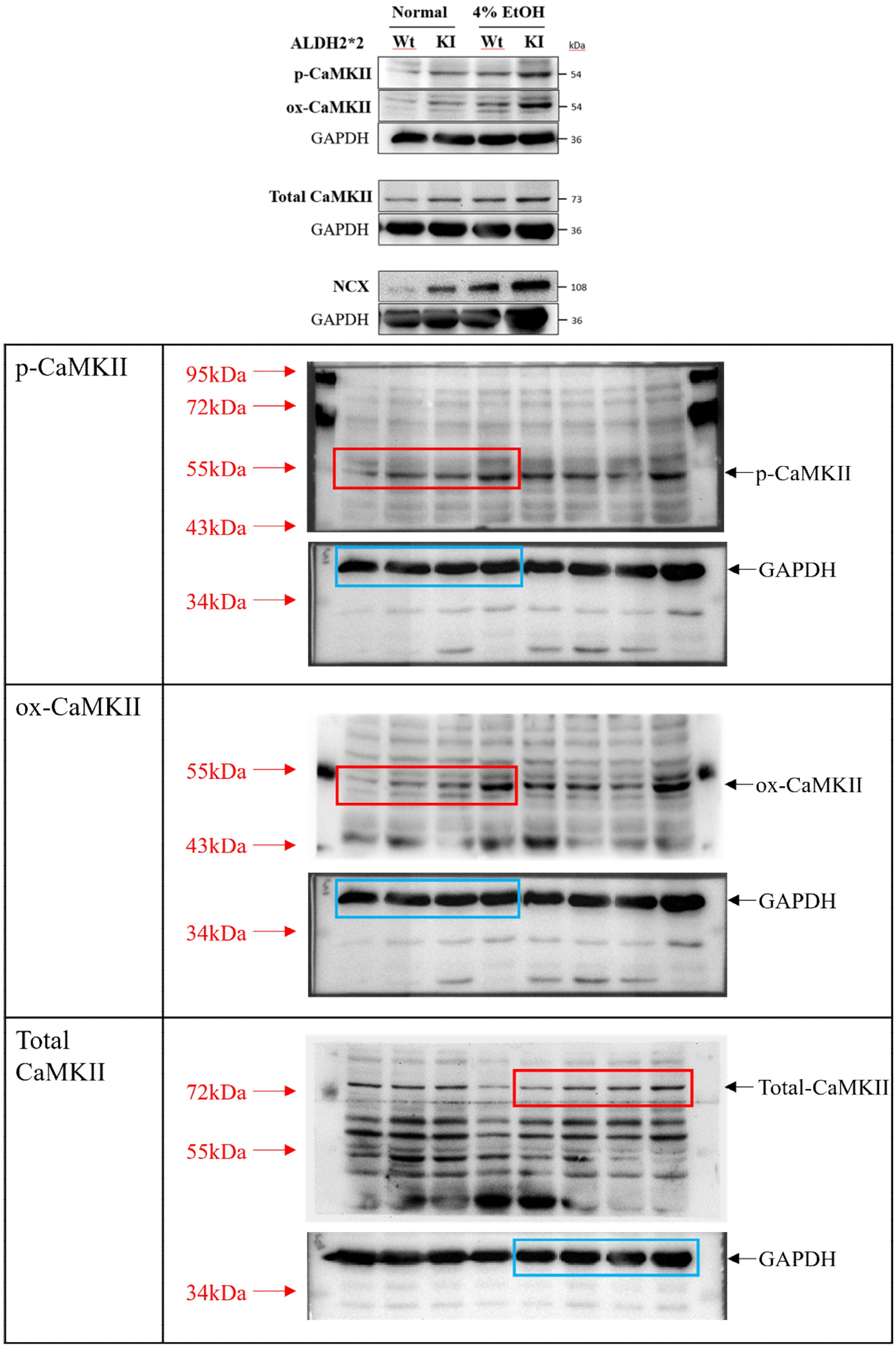

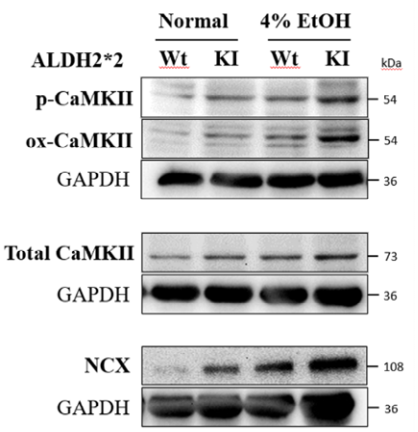


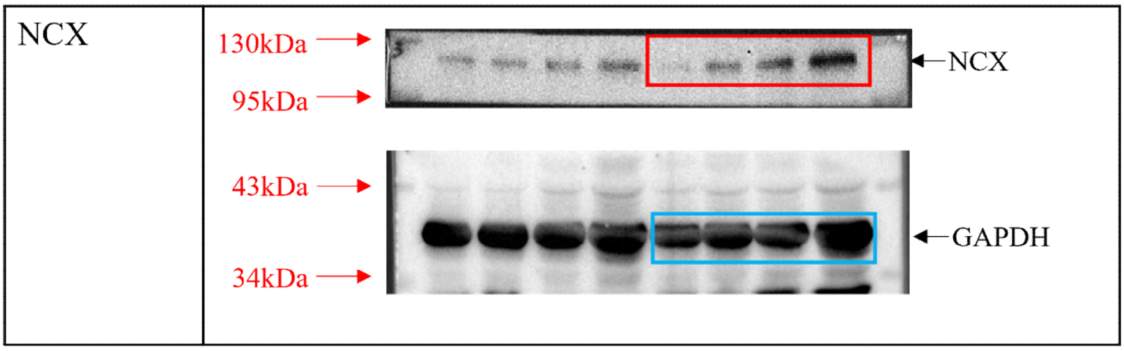


**Supplementary Figure 15. Uncropped original densitometric western blot analysis of heart tissue for CaMKII (total, phosphorylated [p-CaMKII] and oxidized [ox-CaMKII] forms) and NCX.**
