# Supplementary material for: A Common East Asian aldehyde dehydrogenase 2*2 variant promotes ventricular arrhythmia with chronic light-to-moderate alcohol use in mice
Source: Commun Biol. 2023 Jun 6;6:610. doi: 10.1038/s42003-023-04985-x (PMC10244406; doi:10.1038/s42003-023-04985-x)
Supplement: Supplementary file 2 — Description of Additional Supplementary Files [file 42003_2023_4985_MOESM2_ESM.pdf]

Description of Additional Supplementary Files

File name: Supplementary Movie 1.

Description: Example of reentrant activity during ventricular arrhythmia (VA) in 4% EtOH-treated *ALDH2*2* KI mice. VA, ventricular arrhythmia.

File name: Supplementary Movie 2.

Description: Example of electrical activity during ventricular arrhythmia (VA) in Wt_4% EtOH. VA, ventricular arrhythmia.

File name: Supplementary Movie 3.

Description: Example of electrical activity after programmed electrical stimulation (PES) induction in normal diet Wt mice. PES, programmed electrical stimulation.

File name: Supplementary Movie 4.

Description: Example of electrical activity after programmed electrical stimulation (PES) induction in normal diet *ALDH2*2* KI mice. PES, programmed electrical stimulation.

File name: Supplementary Data 1

Description: Source data underlying figures.

Supplementary Data 1

Source data underlying figures
