# Supplementary material for: A Common East Asian aldehyde dehydrogenase 2*2 variant promotes ventricular arrhythmia with chronic light-to-moderate alcohol use in mice
Source: Commun Biol. 2023 Jun 6;6:610. doi: 10.1038/s42003-023-04985-x (PMC10244406; doi:10.1038/s42003-023-04985-x)
Supplement: Supplementary file 8 — Reporting Summary [file 42003_2023_4985_MOESM8_ESM.pdf]

nature portfolio

Corresponding author(s): Chung-Lieh Hung, MD, MSc, Ph.D; Hung-I Yeh, MD, Ph.D

Last updated by author(s): 2022/3/3

Reporting Summary

Nature Portfolio wishes to improve the reproducibility of the work that we publish. This form provides structure for consistency and transparency in reporting. For further information on Nature Portfolio policies, see our Editorial Policies and the Editorial Policy Checklist.

Statistics

For all statistical analyses, confirm that the following items are present in the figure legend, table legend, main text, or Methods section.

n/a	Confirmed	
☐	☒	The exact sample size (*n*) for each experimental group/condition, given as a discrete number and unit of measurement
☐	☒	A statement on whether measurements were taken from distinct samples or whether the same sample was measured repeatedly
☐	☒	The statistical test(s) used AND whether they are one- or two-sided<br>*Only common tests should be described solely by name; describe more complex techniques in the Methods section.*
☐	☒	A description of all covariates tested
☐	☒	A description of any assumptions or corrections, such as tests of normality and adjustment for multiple comparisons
☐	☒	A full description of the statistical parameters including central tendency (e.g. means) or other basic estimates (e.g. regression coefficient) AND variation (e.g. standard deviation) or associated estimates of uncertainty (e.g. confidence intervals)
☐	☒	For null hypothesis testing, the test statistic (e.g. *F*, *t*, *r*) with confidence intervals, effect sizes, degrees of freedom and *P* value noted<br>*Give P values as exact values whenever suitable.*
☒	☐	For Bayesian analysis, information on the choice of priors and Markov chain Monte Carlo settings
☒	☐	For hierarchical and complex designs, identification of the appropriate level for tests and full reporting of outcomes
☒	☐	Estimates of effect sizes (e.g. Cohen's *d*, Pearson's *r*), indicating how they were calculated

Our web collection on statistics for biologists contains articles on many of the points above.

Software and code

Policy information about availability of computer code

Data collection	*Provide a description of all commercial, open source and custom code used to collect the data in this study, specifying the version used OR state that no software was used.*
Data analysis	*Provide a description of all commercial, open source and custom code used to analyse the data in this study, specifying the version used OR state that no software was used.*

For manuscripts utilizing custom algorithms or software that are central to the research but not yet described in published literature, software must be made available to editors and reviewers. We strongly encourage code deposition in a community repository (e.g. GitHub). See the Nature Portfolio guidelines for submitting code & software for further information.

Data

Policy information about availability of data

All manuscripts must include a data availability statement. This statement should provide the following information, where applicable:

- Accession codes, unique identifiers, or web links for publicly available datasets
- A description of any restrictions on data availability
- For clinical datasets or third party data, please ensure that the statement adheres to our policy

The datasets generated during and/or analysed during the current study are available from the corresponding author on reasonable request.

Field-specific reporting

Please select the one below that is the best fit for your research. If you are not sure, read the appropriate sections before making your selection.

☒ Life sciences ☐ Behavioural & social sciences ☐ Ecological, evolutionary & environmental sciences

For a reference copy of the document with all sections, see nature.com/documents/nr-reporting-summary-flat.pdf

Life sciences study design

All studies must disclose on these points even when the disclosure is negative.

Sample size	In our previous study conducted in prospective manner, a sample size of totally 237 human participants is enough for significant statistical differences regarding baseline characteristics and pre-specified cardiac structural (e.g., cardiac dimension) and electrophysiological differences (e.g., electrocardiography [ECG]) with same exposed risk factor of alcohol use.
Data exclusions	8 enrolled participants were excluded due to incomplete genetic information; 4 and 3 further excluded due to incomplete baseline demographic information and heavy alcohol use, which did not fulfill our study criteria.
Replication	Physiological testing results were reproducible from our laboratory and have been published else where with similar topics and methods used.
Randomization	Study participants were categorized according to exposed daily alcohol use (non-users or habitual users). Co-variates were further adjusted based on empirical clinical confounders that may influence our measures or outcomes assessed.
Blinding	During data collection, physiological measures (e.g., cardiac structure and electrophysiological indices) were acquired by technicians or laboratory staff blinded to study design.

Reporting for specific materials, systems and methods

We require information from authors about some types of materials, experimental systems and methods used in many studies. Here, indicate whether each material, system or method listed is relevant to your study. If you are not sure if a list item applies to your research, read the appropriate section before selecting a response.

Materials & experimental systems

n/a	Involved in the study	
☐	☒	Antibodies
☒	☐	Eukaryotic cell lines
☒	☐	Palaeontology and archaeology
☐	☒	Animals and other organisms
☐	☒	Human research participants
☒	☐	Clinical data
☒	☐	Dual use research of concern

Methods

n/a	Involved in the study	
☒	☐	ChIP-seq
☒	☐	Flow cytometry
☒	☐	MRI-based neuroimaging

Antibodies

Antibodies used	Collagen-1 (1:1000, mouse monoclonal, Sigma), Cx43 (1:250, mouse monoclone, BD Biosciences), Cx43 (1:1000, rabbit polyclone, Sigma-Aldrich) to recognize the phosphorylated (functional) (p-Cx43) form and non-phosphorylated (np-Cx43 [Cx43-P0]) isoforms of Cx43 as previously described (3). TGF-beta 1(1:500, mouse monoclone, Santa Cruz), Nav1.5 (1:200, rabbit polyclonal clone, Alomone Labs), Kv1.4 (1:200, rabbit polyclonal clone, Alomone Labs), Kv4.2 (1:200, rabbit polyclone, Alomone Labs), Kv4.3 (1:200, rabbit polyclone, Alomone labs), Cav1.2 (1:250, rabbit polyclone, Alomone Labs), Cav1.3 (1:250, mouse monoclone, Gene Tex), NCX1 (1:1000, mouse monoclone, Gene Tex), p (Thr286)- Calcium/calmodulin-dependent protein kinase II (CaMKII) (1:2000, mouse monoclonal, Gene Tex) , ox-CaMKII (1:1000, rabbit polyclonal clone, Gene Tex), and Total CaMKII (1:1000, rabbit polyclonal clone, Gene Tex)
Validation	1. Mouse monoclonal anti-Collagen type 1 (1:1000, Sigma-Aldrich, https://www.sigmaaldrich.com/TW/en/product/sigma/c2456?gclid=Cj0KCQiA64GRBhCZARIsAHOLriJg6a_FH4sILTVVX3lhXcP0_rNcxtQbkQw2otUez5waDTWEAcT0kkwaAucDEALw_wcB).<br>2. Mouse monoclonal anti-Cx43 (1:250, BD Biosciences, https://www.bdbiosciences.com/en-us/products/reagents/microscopy-imaging-reagents/immunofluorescence-reagents/purified-mouse-anti-connexin-43.610062).<br>3. Rabbit polyclonal anti-Cx43 (1:1000, Sigma-Aldrich, https://www.sigmaaldrich.com/TW/en/product/sigma/c6219).<br>4. Mouse monoclonal anti-TGF-beta 1(3C11) (1:500, Santa Cruz, https://www.scbt.com/p/tgf-beta1-antibody-3c11).<br>5. Rabbit polyclonal anti-Nav1.5 (SCN5A) (493-511) (1:200, Alomone Labs, https://www.alomone.com/p/anti-nav1-5/ASC-005?gclid=Cj0KCQiA64GRBhCZARIsAHOLriKWytgxwbHJIajK86LceMagh3yDaK62yGbstqO9zrPiUsLfiDa9G-MaAiYBEALw_wcB).<br>6. Rabbit polyclonal anti-Kv1.4 (1:200, Alomone Labs, https://www.alomone.com/p/anti-kv1-4/APC-007).<br>7. Rabbit polyclonal anti-Kv4.2 (1:200, Alomone Labs, https://www.alomone.com/p/anti-kv4-2/APC-023).<br>8. Rabbit polyclonal anti-Kv4.3 (1:200, Alomone Labs, https://www.alomone.com/p/anti-kv4-3/APC-017).

9. Rabbit polyclonal anti-Cav1.2 (CACNA1C) (1:250, Alomone Labs, https://www.alomone.com/p/anti-cav1-2-antibody/ACC-003).
10. Mouse monoclonal anti-Cav1.3 (1:250, Gene Tex, https://www.genetex.com/Product/Detail/Cav1-3-antibody-S48A-9/GTX41982).
11. Mouse monoclonal anti-NCX1 [C2C12](1:1000, Gene Tex, https://www.genetex.com/Product/Detail/NCX1-antibody-C2C12/GTX22869).
12. Mouse monoclonal anti-CaMKII alpha (phospho Thr286) (1:2000, Gene Tex, https://www.genetex.com/Product/Detail/CaMKII-alpha-phospho-Thr286-antibody-22B1/GTX22724).
13. Rabbit polyclonal anti-CaMKII (oxidized) (1:1000, Gene Tex, https://www.genetex.com/Product/Detail/CaMKII-oxidized-antibody/GTX36254).
14. Rabbit polyclonal anti-CaMKII (1:1000, Gene Tex, https://www.genetex.com/Product/Detail/CaMKII-antibody/GTX135117).

Animals and other organisms

Policy information about studies involving animals; ARRIVE guidelines recommended for reporting animal research

Laboratory animals	4-month ALDH2*2 KI animal study complied with the institutional and national guidelines for the care and use of laboratory animals (Taiwan Animal Protection Law; Scientific Application of Animals, 1998). All animals used in this study (homozygous ALDH2*2 mice and their wild-type littermates) had a C57BL/6 genetic background, and the protocols were reviewed by the institutional board (IACUC:MMH-A-S-107-69). Genotypes of mice were determined via polymerase chain reaction (PCR) of tail tip DNA (Supplementary materials). The construction and genotype of ALDH2*2 knock-in mice have been described previously [19]. Four-month-old wild type (WT) male C57BL/6 mice or homozygous ALDH2*2 knock-in (ALDH2*2 KI) mice were assigned into two diet groups as described in a previously published protocol of ours [16]. These were, (i) the normal diet group (ad libitum liquid diet; dry mix #F1259SP Bio-Serv® Advancing Science. Enriching Animals, USA Test with warm tap water); and (ii) the 4% EtOH group (4% v/v alcohol liquid diet; dry mix #F1697SP, Bio-Serv® supplemented with maltose dextrin, ethanol, and warm tap water).
Wild animals	C57BL/6JNarl was provided by National Laboratory Animal Center (NLAC), NARLabs, Taiwan.
Field-collected samples	The mice were housed at 22–24 °C with light: dark cycles of 12:12 hours at animal center of MacKay Memorial Hospital.
Ethics oversight	The experimental protocol was approved by the Animal Care and Use Committee from the Institutional Review Board of MacKay Memorial Hospital.

Note that full information on the approval of the study protocol must also be provided in the manuscript.

Human research participants

Policy information about studies involving human research participants

Population characteristics	Among the 196 adults with habitual light-to-moderate alcohol consumption (median: 13.9 [7.6 ~ 29.3] g/day, 1.2 [0.6~2.4] standard drinks/day), 59 (30.1%) carried the ALDH2 rs671 variant allele ([ALDH2 Vt]; G/A or A/A,) while 137 (69.9%) were ALDH2 wild type ([ALDH2 Wt]; G/G, Table 1). Among the 57 alcohol non-users, 28 (49.1%) were ALDH2 Vt and 29 (50.9%) were ALDH2 Wt.
Recruitment	Study participants were prospectively recruited. Physiological studies including 12-lead body surface electrocardiogram (ECG) results, anthropometrics (such as body height, weight, and waist circumferences), biochemical information (including lipid profiles and renal function defined as eGFR using the MDRD formula), and background medical histories were carried out or obtained by blinded nursing/technicians. Acquisition or quantification of these measures were conducted in a blinded manner. All our study participants provided informed consent regarding participation of this study.
Ethics oversight	This study passed the Institutional Review Board of MacKay Memorial Hospital (14MMHIS069) and complied with the Declaration of Helsinki.

Note that full information on the approval of the study protocol must also be provided in the manuscript.
